# Concanavalin A targets phylogenetically conserved N-linked glycans on coronavirus spike proteins for broad-spectrum antiviral activity

**DOI:** 10.1128/jvi.01679-25

**Published:** 2026-04-27

**Authors:** Dekuan Guo, Shi Yu, Kaixiong Ma, Hua Tao, Qingxing Wang, Sirui Han, Qiangyun Ai, Huina Hu, Xiancai Ma, Geng Li, Shaobo Wang

**Affiliations:** 1 State Key Laboratory of Traditional Chinese Medicine Syndrome, Guangzhou University of Chinese Medicine 47879, Guangzhou, China; 2 Department of Basic Research, Guangzhou International Bio-Island, Guangzhou National Laboratory 612039, Guangzhou, China; 3 State Key Laboratory of Respiratory Disease, Guangzhou Medical University, The First Affiliated Hospital of Guangzhou Medical University, Guangzhou National Laboratory Clinical Base, School of Basic Medical Sciences 655509, Guangzhou, China; 4 Wuhan Institute of Virology, Chinese Academy of Sciences 74614 , Wuhan, China; 5 University of the Chinese Academy of Sciences, Beijing, China; University of North Carolina at Chapel Hill, Chapel Hill, North Carolina, USA

**Keywords:** coronavirus, viral entry, spike, glycoprotein, lectin, N-linked glycosylation, antiviral compound, membrane fusion

## Abstract

The rapid evolution of SARS-CoV-2 variants, driven by antigenic drift in the spike glycoprotein, continues to undermine the efficacy of current vaccines and monoclonal antibody therapies. Targeting conserved features of the spike protein has been a major focus against coronavirus entry and the development of therapeutics. Here, we demonstrate that the plant lectin concanavalin A (ConA) broadly inhibits coronavirus entry through a conserved mechanism. With a combination of cell-cell fusion, pseudoviral entry, and authentic virus infection models, we show that ConA broadly inhibits coronavirus spike-mediated membrane fusion and viral entry. Biochemical analyses reveal that ConA targets two highly conserved N-glycosylation sites outside the receptor binding domain, flanking the S2′ cleavage site via its mannose-binding properties. This interaction sterically impedes proteolytic activation of the spike, a molecular step essential for membrane fusion. ConA exhibited nanomolar efficacy against hCoV-NL63 infections *in vitro* and significantly reduced viral load and mitigated lung pathology in hCoV-NL63-infected mice *in vivo*. Our findings reveal specific N-linked glycosylation sites as a major vulnerability of the spike and highlight ConA as a prototype for the development of lectin-based therapeutics against emerging coronavirus infections.

**IMPORTANCE:**

The rapid evolution of SARS-CoV-2 variants, which evade current vaccines and therapeutics by altering epitopes on the spike protein, highlights a critical need for broad-spectrum antivirals. This study investigates concanavalin A (ConA), a legume lectin that targets highly conserved N-linked glycosylation sites on the spike protein, as a potential pan-coronavirus entry inhibitor. ConA broadly inhibits diverse coronaviruses by blocking spike-mediated membrane fusion. In contrast to previously reported antiviral lectins, ConA binds specifically to high-mannose oligosaccharides by targeting two phylogenetically conserved residues in the S2 subunit outside the receptor-binding domain. Consequently, ConA binding prevents the proteolytic activation of S2′ and effectively inhibits membrane fusion and coronavirus infection both *in vitro* and *in vivo*. This work identifies conserved N-glycosylation sites on the spike protein as stable, vulnerable targets for antiviral intervention, distinct from the variable epitopes recognized by antibodies. These findings indicate that lectins like ConA may provide a promising approach for developing effective antivirals against emerging coronaviruses.

## INTRODUCTION

Variants of severe acute respiratory syndrome coronavirus 2 (SARS-CoV-2) challenge currently available vaccines and therapeutics through epitope change on the viral spike glycoprotein (1). This accelerating antigenic evolution necessitates the urgent development of next-generation antivirals that target conserved structural motifs or entry mechanisms to achieve pan-coronavirus inhibition. As a result, understanding evolutionarily conserved vulnerabilities in the spike protein remains a major research priority for developing broad-spectrum interventions against coronavirus infections.

Spike-mediated SARS-CoV-2 entry involves two essential steps. First, the spike S1 subunit contains a receptor-binding domain (RBD) that recognizes the host cell angiotensin-converting enzyme 2 (ACE2) expressed on the host cell membrane, triggering allosteric changes within the spike protein (2–5). Next, the functionally rearranged S2 subunit requires proteolytic processing at the S2′ cleavage site by the host proteases, either by TMPRSS2 localized on the plasma membrane or cathepsins within the endosomal compartment (6–8). Spike proteolytic activation serves to expose the fusion peptide, enabling its docking with the host cell membrane. The membrane fusion process is ultimately achieved through refolding of the heptad repeat 1 (HR1) and HR2 domains, which physically pulls membranes into proximity (9, 10). Our previous work demonstrated that the S2′ proteolytic cleavage as well as accessible cholesterol on the plasma membrane is essential for spike-mediated membrane fusion (11), and it can be regulated through the expression of the ACE2 receptor or proteases such as TMPRSS2 (12). Thus, the development of antiviral drugs and prophylactics against the fusion machinery may broadly and efficiently block the spike-mediated membrane fusion and subsequent viral entry.

Apart from the molecular event during the coronavirus entry process, N-linked glycans decorated on the mature spike protein are a conserved molecular feature across various coronaviruses (13). The spike trimers display significantly higher density of N-linked glycosylation modification compared to analogous modifications on the host cell membrane proteins (14). All coronavirus spike proteins harbor distinct N-linked glycosylation sites exposed outside the transmembrane domain and covering approximately 30% epitopes on the trimeric structure (15). The oligomannosidic-type glycans not only facilitate the proper post-translational folding but also ensure the half-life and intracellular trafficking of the spike protein, ultimately supporting its membrane localization and virion assembly (16). The complex N-linked modifications further create highly branched glycans with enhanced topological complexity, which critically modulate viral-host cell affinity (17) and subcellular targeting, and may play a role in antibody evasion (18–21).

Lectins are proteins that selectively bind to carbohydrates. Xenogeneic antiviral lectins not only retain binding capacity to viral glycoproteins but also avoid activating human cellular signaling pathways, thereby circumventing potential off-target effects (22, 23). Plant-derived lectins evolved as defense mechanisms against phytopathogens, analogous to antibodies produced by the adaptive immune system in humans. Among the earliest discovered plant lectins, engineered banana lectin (BanLec) demonstrates remarkable anti-coronavirus activity, showing inhibitory effects against live Middle East respiratory syndrome coronavirus (MERS-CoV) in cell cultures and SARS-CoV-2 in hamster infection models (24). Griffithsin, a red algal lectin, also exhibits broad-spectrum anti-viral activity against the SARS-CoV-2 (25). The dimeric form of griffithsin binds to the S2 subunit of the spike protein and demonstrates superior inhibitory efficacy against SARS-CoV-2 pseudovirus entry compared to the HR2 mimetic inhibitor EK1 (26). Although these lectins have been shown to bind SARS-CoV-2 spikes, their molecular modes of action targeting glycosylation sites have not been experimentally validated.

Compared to BanLec and griffithsin, legume lectins possess natural oligomerization properties and unique binding modalities (27). Native legume lectins adopt stable tetrameric, pentameric, or octameric configurations, achieving enhanced avidity through at least four binding pockets per tetramer. For instance, the *Canavalia ensiformis* (jack bean) lectin concanavalin A (ConA) maintains tetrameric stability under physiological pH and ambient temperatures (28), although its suitability for respiratory tract applications requires further exploration. A previous study has demonstrated that ConA and other legume lectins display carbohydrate binding to the purified spike ectodomain (14). Binding of ConA and other plant-derived lectins to the spike can lead to the agglutination of SARS-CoV-2 viral particles, leading to the loss of viral infectivity (29). However, the functional outcome of spike-ConA binding on membrane fusion and the molecular mechanism of ConA-inhibited SARS-CoV-2 viral entry has not been defined.

Based on a cell-cell fusion model, we have previously demonstrated that the binding of ACE2 to the SARS-CoV-2 spike protein primes the cleavage at the S2′ site adjacent to the fusion peptide (FP) region (8, 30). This cleaved S2′ species is essential for driving the membrane fusion. We hypothesize that legume lectins, such as ConA, may exert specific antiviral effects at multiple stages of spike activation. For instance, ConA could target critical N-linked glycosylation sites within the RBD, thereby sterically hindering the viral attachment to the host receptor. Alternatively, the binding of oligomeric ConA could spatially regulate the proteolytic activation of the S2′, blocking the release of the FP and subsequent interactions with the host cell membrane. Additionally, downstream of the fusion peptide release, ConA may impede the refolding of the heptad repeat (HR) domains, thus disrupting the structural rearrangements essential for membrane fusion. Given the potential of ConA to interfere with multiple stages of SARS-CoV-2 spike protein activation, including viral attachment, proteolytic cleavage, and membrane fusion, a closer investigation of ConA is warranted to explore its therapeutic antiviral potential.

Here, we characterize the antiviral effect of ConA in the SARS-CoV-2 spike-mediated cell-cell fusion and pseudovirus entry models. We demonstrate that ConA serves as a broad-spectrum inhibitor of spike-mediated cell-cell fusion and pseudovirus entry, not only across various SARS-CoV-2 spike variants but also for spikes from other coronaviruses. Biochemical analysis reveals that ConA physically interacts with the spike through recognizing two conserved N-glycosylation sites, adjacent to the S2′ cleavage site, and distal to the RBD. This interaction impedes the proteolytic activation of spike protein, thereby preventing subsequent spike-mediated membrane fusion. *In vitro* studies show that ConA displays excellent antiviral activities against authentic hCoV-NL63 infections. Furthermore, intranasal administration of ConA in a murine model of hCoV-NL63 infection effectively suppresses viral replication and alleviates lung pathology. Collectively, these results highlight that conserved spike N-linked glycosylation sites are susceptible to antiviral legume lectins and ConA as an effective intervention against potential future coronavirus threats.

## RESULTS

### Concanavalin A inhibits SARS-CoV-2 spike-mediated membrane fusion and pseudoviral entry

To identify potential plant-derived lectins binding to the SARS-CoV-2 spike, we initially developed an indirect enzyme-linked immunosorbent assay (ELISA) to screen for putative binders to the recombinant pre-fusion spike ectodomains. A panel of plant-derived lectins was coated onto the ELISA plate based on their selective binding characteristics to distinct sugar moieties. These include the galactose-specific peanut agglutinin (PNA), sialic acid- and glucose-specific wheat germ agglutinin (WGA), and oligomannose-specific concanavalin A (ConA) (Fig. S1A). We found that only ConA exhibited the highest binding to the SARS-CoV-2 prefusion spike ectodomain (Fig. S1B), followed by WGA and PHA displaying much weaker binding to the SARS-CoV-2 spike ectodomain at similar concentrations.

**Fig 1:**
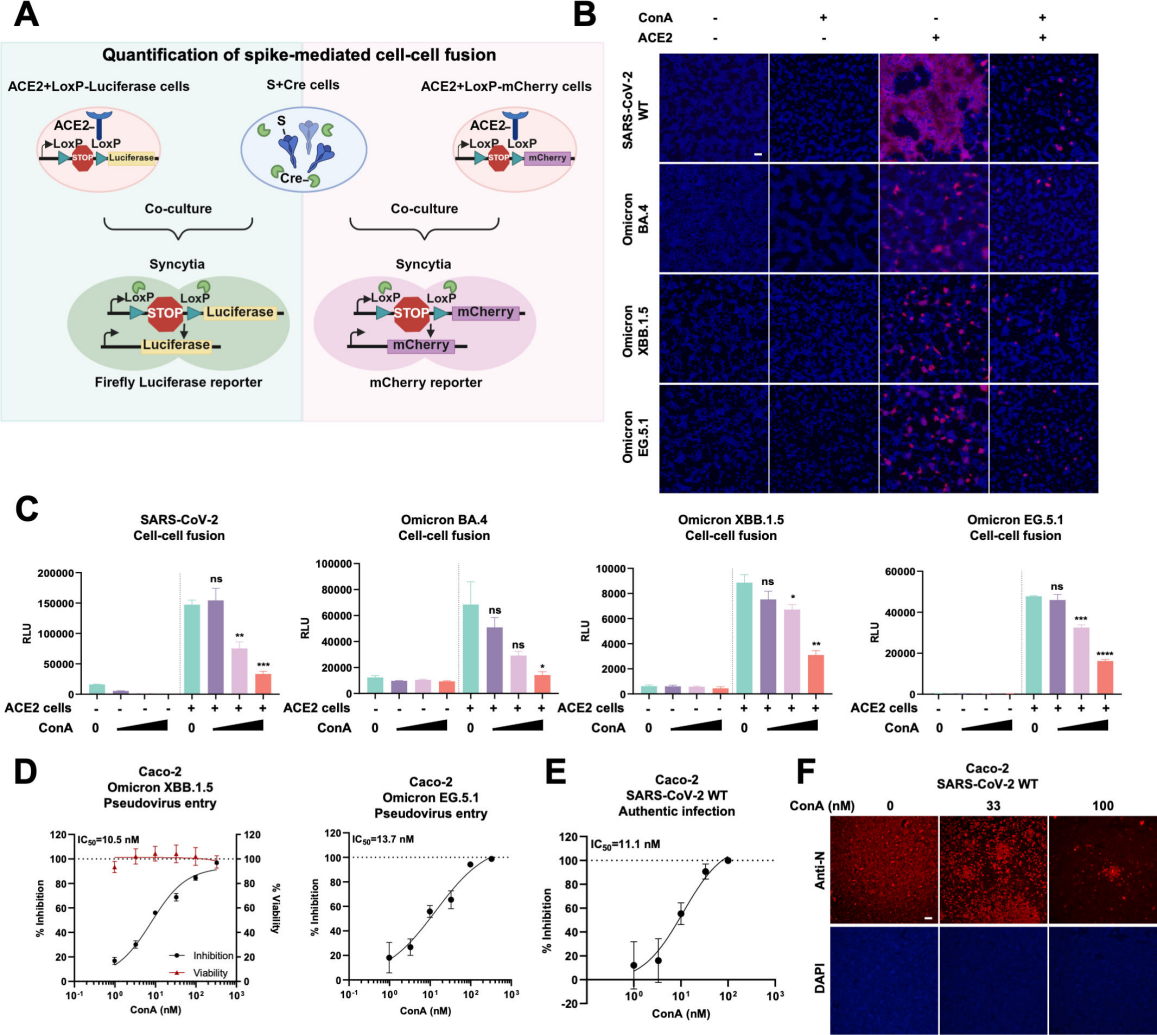
Concanavalin A broadly inhibits SARS-CoV-2 spike-mediated membrane fusion and viral entry. (**A**) Schematics of the cell-cell fusion co-culture model used to quantify or qualify spike-mediated syncytium formation. Cells co-expressing SARS-CoV-2 spike and Cre were co-cultured with LoxP-luc or LoxP-mCherry expressing HEK293T cells for 16 h, before syncytia formation was captured using the fluorescence derived from mCherry reporter or quantified based on bioluminescence signal derived from the firefly luciferase. Different concentrations of Concanavalin A (ConA) were added at the time of co-culture. (**B**) Representative fluorescent images of mCherry reporter and nuclei counterstained with 100 ng/mL Hoescht33342 collected from SARS-CoV-2 WT and SARS-CoV-2 variants (Omicron BA.4, XBB.1.5, and EG.5.1) spike-expressing cells, co-cultured with LoxP-mCherry control HEK293T or HEK293T-ACE2 cells for 16 h in the absence or presence of 10 µg/mL ConA. The scale bar is representative of 100 μm. (**C**) Luciferase activity (RLU) detected from WT, Omicron BA.4, XBB.1.5, or EG.5.1 spike-expressing cells, co-cultured with LoxP-luciferase control HEK293T or HEK293T-ACE2 cells for 16 h in the absence or presence of 19.60, 65.29, and 196.07 nM (2, 6.6, and 20 µg/mL) ConA. Data are representative of three repeats, and data points are represented as mean ± SEM. Statistical significance was determined using one-way ANOVA with Sidak post-hoc multiple comparison. *P* values are indicated as ns, not significant, *P < 0.05, **P < 0.005, ***P < 0.0005, ****P < 0.0001. (**D**) The IC_50_ curve (black) and cytotoxicity (red) of ConA against Omicron XBB.1.5 and EG.5.1 spike variants pseudovirus entry, respectively, in Caco-2 cells. Data are representative of three repeats. (**E**) Normalized IC_50_ curve of ConA-mediated inhibition of SARS-CoV-2 WT viral copies in the infected Caco-2 cells supernatant at MOI = 0.01 for 24 h. Viral copies were determined using quantitative PCR targeting the SARS-CoV-2 E genes. (**F**) Representative fluorescent images of SARS-CoV-2 nucleocapsid (N) and nuclei counterstained with 1 μg/mL DAPI, captured from Caco-2 cells infected with SARS-CoV-2 (MOI = 0.01) in the absence or presence of 32.65 and 98.04 nM ConA for 24 h. The scale bar is representative of 100 μm.

More importantly, to investigate the direct antagonistic effect of exogenous concanavalin A (ConA) on the viral spike protein, we employed a Cre/LoxP-based co-culture system. This system allows for both quantitative and qualitative measurements of spike-mediated membrane fusion, either in the absence or presence of exogenously added lectin, as detected by firefly luciferase activity and mCherry fluorescence, respectively (31, 32) (Fig. 1A). When HEK293T cells co-expressing WT, BA.4, XBB.1.5, and E.G.5.1 spike proteins along with Cre recombinase were co-cultured for 16 h with either LoxP-mCherry-transfected control HEK293T cells or HEK293T cells stably expressing human ACE2 (HEK293T-ACE2), robust fluorescent images of the mCherry reporter in syncytia can be captured specifically from the HEK293T-ACE2 groups. More importantly, the addition of 196 nM (20 µg/mL) ConA into the cell supernatants at the beginning of the co-culture assay significantly reduced cell-cell fusion between cells expressing the ancestral spike and HEK293T-ACE2 cells (Fig. 1B). When Cre and spike-expressing cells were co-cultured with the LoxP-luciferase reporter HEK293T-ACE2 cells, quantitative bioluminescence readings showed that ConA displayed dose-dependent inhibition against the SARS-CoV-2 WT spike-induced cell-cell fusion (Fig. 1C). Although Omicron BA.4, XBB.1.5, and E.G.5.1 spikes exhibited reduced fusogenicity when co-cultured with ACE2 cells (33, 34), the inhibitory effect of ConA on cell-cell fusion remained consistent across these subvariants (Fig. 1C). These results suggested that ConA directly modulates spike-mediated membrane fusion across various SARS-CoV-2 spike variants.

To investigate the effect of exogenous ConA on SARS-CoV-2 pseudovirus entry, we pre-incubated vesicular stomatitis virus (VSV) pseudovirus bearing the SARS-CoV-2 WT spike with serially diluted ConA and then infected HEK293T cells stably expressing human ACE2 (HEK293T-ACE2) or Caco-2 cells. According to half-logarithmic dilutions of ConA, the half-maximal inhibitory concentrations (IC_50_) of ConA-mediated inhibition on pseudovirus entry on Caco-2 cells are 10.6 nM and 13.7 nM for Omicron XBB.1.5 and EG.5.1 spike variants, respectively (Fig. 1D, Left). At an effective dose up to 196 nM, ConA displayed no cytotoxicity in Caco-2 cells up to 24 h (Fig. 1D). Moreover, the IC_50_ of ConA is 8.7 nM for the Omicron XBB.1.5 spike variant and 9.1 nM for the EG.5.1 spike variant pseudovirus entry on HEK293T-ACE2 cells (Fig. S1D). At a concentration of 98.04 nM, ConA significantly inhibited the entry of SARS-CoV-2 WT, Omicron XBB.1.5, EG.5.1, and BA.4 spike-mediated pseudoviruses by more than 80% (Fig. S1E). At concentrations (9.804–98.04 nM) effective for inhibiting the SARS-CoV-2 pseudovirus entry and cell-cell fusion, ConA treatment had no cytotoxicity or confluency of either Caco-2 or HEK293T cells (Fig. S1F). Thus, ConA broadly inhibited SARS-CoV-2 spike-mediated cell-cell fusion and pseudovirus entry, independent of spike variants.

### Concanavalin A exhibits broad-spectrum inhibitory activities against spikes from other coronaviruses

**Fig 2:**
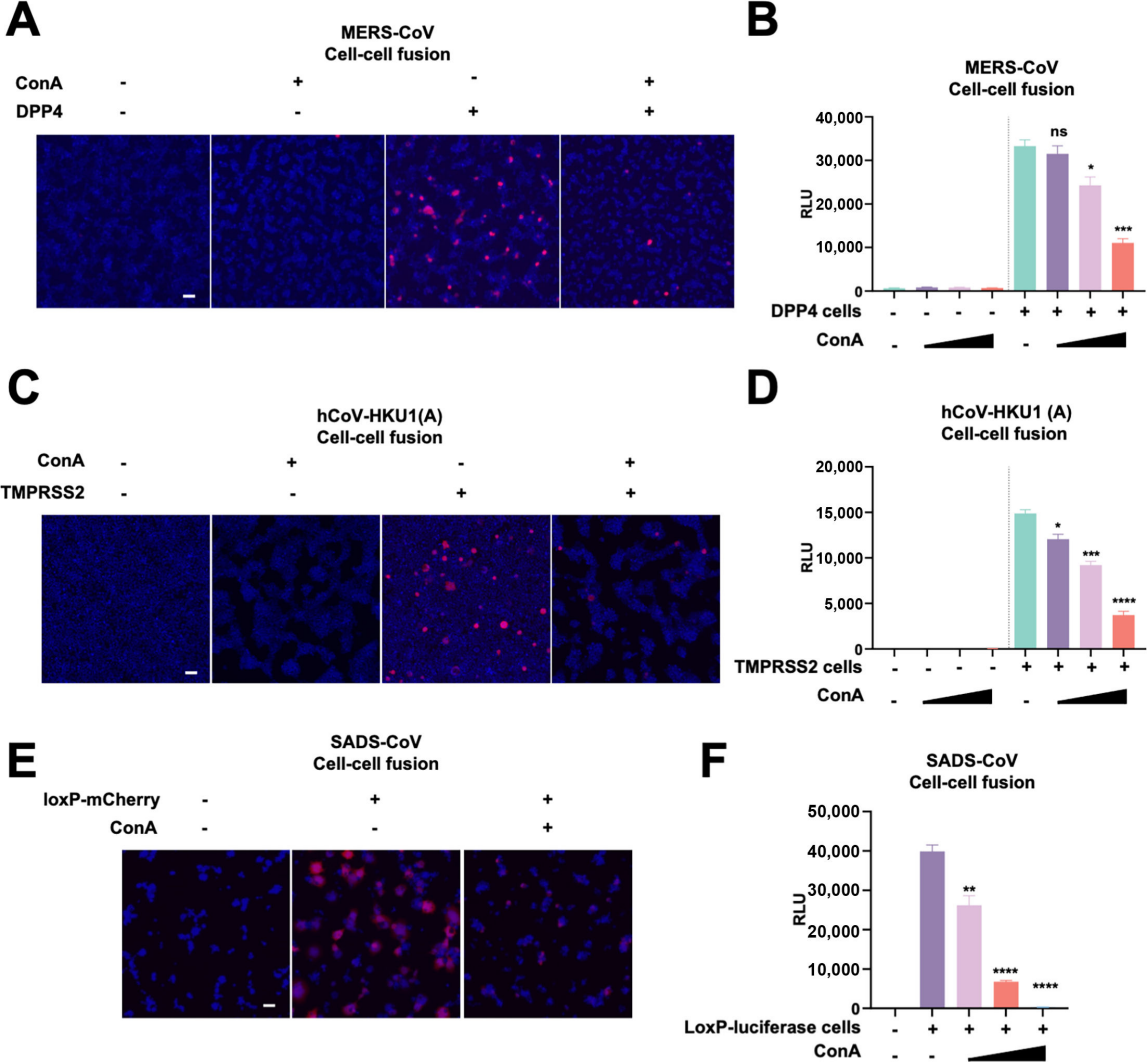
Concanavalin A exhibits broad-spectrum inhibitory activities against spikes from other coronaviruses. (**A**) Representative fluorescent images of mCherry reporter and nuclei counterstained with 100 ng/mL Hoescht 33342 collected from cells expressing the MERS-CoV spike and Cre, co-cultured with LoxP-mCherry control HEK293T or HEK293T cells overexpressing DPP4 for 16 h in the absence or presence of 196.07 nM (20 µg/mL) ConA. The scale bar is representative of 100 μm. (**B**) Luciferase activity (RLU) detected from cell lysates of Cre and MERS-CoV spike-expressing cells, co-cultured with LoxP-luciferase control HEK293T or HEK293T overexpressing DPP4 cells for 16 h in the absence or presence of 19.60, 65.29, and 196.07 nM (2, 6.6, and 20 µg/mL) ConA. (**C**) Representative fluorescent images of mCherry reporter and nuclei counterstained with 100 ng/mL Hoescht 33342 collected from cells expressing the hCoV-HKU1 (A) spike and Cre, co-cultured with LoxP-mCherry control HEK293T or HEK293T cells overexpressing TMPRSS2 for 16 h in the absence or presence of 196.07 nM ConA. The scale bar is representative of 100 μm. (**D**) Luciferase activity (RLU) detected from cell lysates of Cre and MERS-CoV spike-expressing cells, co-cultured with LoxP-luciferase control HEK293T or HEK293T overexpressing DPP4 cells for 16 h in the absence or presence of 19.60, 65.29, and 196.07 nM ConA. (**E**) Representative fluorescent images of mCherry reporter and nuclei counterstained with 100 ng/mL Hoescht 33342 collected from cells expressing the SADS-CoV spike and Cre, co-cultured with control HEK293T or LoxP-mCherry HEK293T cells for 16 h in the absence or presence of 196.07 nM ConA. The scale bar is representative of 100 μm. (**F**) Luciferase activity (RLU) detected from cell lysates of Cre and MERS-CoV spike-expressing cells, co-cultured with control HEK293T or LoxP-luciferase HEK293T cells for 16 h in the absence or presence of 19.60, 65.29, and 196.07 nM ConA. Data are representative of three repeats, and data points are represented as mean ± SEM. Statistical significance was determined using one-way ANOVA with Sidak post-hoc multiple comparison test. *P* values are indicated as *ns,* not significant, *P < 0.05, **P < 0.005, ***P < 0.0005, ****P < 0.0001.

Since ConA has shown effectiveness against the cell-cell fusion and pseudovirus entry from SARS-CoV-2 spike variants, we further utilized the cell-cell fusion system to test spikes from the middle east respiratory syndrome (MERS-CoV) and human coronavirus HKU1 (hCoV-HKU1) from the β-coronavirus genus and swine acute diarrhea syndrome (SADS-CoV) from the α-coronavirus genus. Similar to the SARS-CoV-2 spike, we were able to quantify the cell-cell fusion between MERS-CoV spike-expressing cells and dipeptidyl peptidase 4 (DPP4) receptor cells either by fluorescence images of syncytia displaying the mCherry reporter (Fig. 2A) or bioluminescence signals produced from the firefly luciferase reporter (Fig. 2B). Treatment with ConA during the co-culture assay also inhibited the MERS-CoV spike-mediated cell-cell fusion in a dose-dependent manner (Fig. 2A and B). TMPRSS2 has been previously identified as a functional receptor for the hCoV-HKU1 (35). Co-culture of hCoV-HKU1 (A) spike-expressing HEK293T cells with HEK293T cells expressing the TMPRSS2 receptor also displayed mCherry reporter and bioluminescence signal due to cell-cell fusion. More importantly, the addition of ConA also dose-dependently inhibited HKU1 spike-mediated cell-cell fusion (Fig. 2C and D). Finally, the receptor for α-coronavirus SADS-CoV spike has yet to be identified. Since the expression of SADS-CoV spike generated robust syncytia formation without transfection of additional receptor, we co-cultured Cre and spike-expressing HEK293T cells with control HEK293T without the reporter transfection, or HEK293T carrying a LoxP-mCherry or LoxP-luciferase cells, to quantify the cell-cell fusion. Expression of SADS-CoV spike results in spontaneous cell-cell fusion in the donor HEK293T cells without the transfection of additional receptors; addition of ConA also dose-dependently inhibited SADS-CoV spike-mediated cell-cell fusion (Fig. 2E and F). These data suggest that ConA exhibits broad-spectrum inhibitory activity against spike-mediated cell-cell fusion in other coronaviruses.

### Concanavalin A directly interacts with spike via N-linked glycans

**Fig 3:**
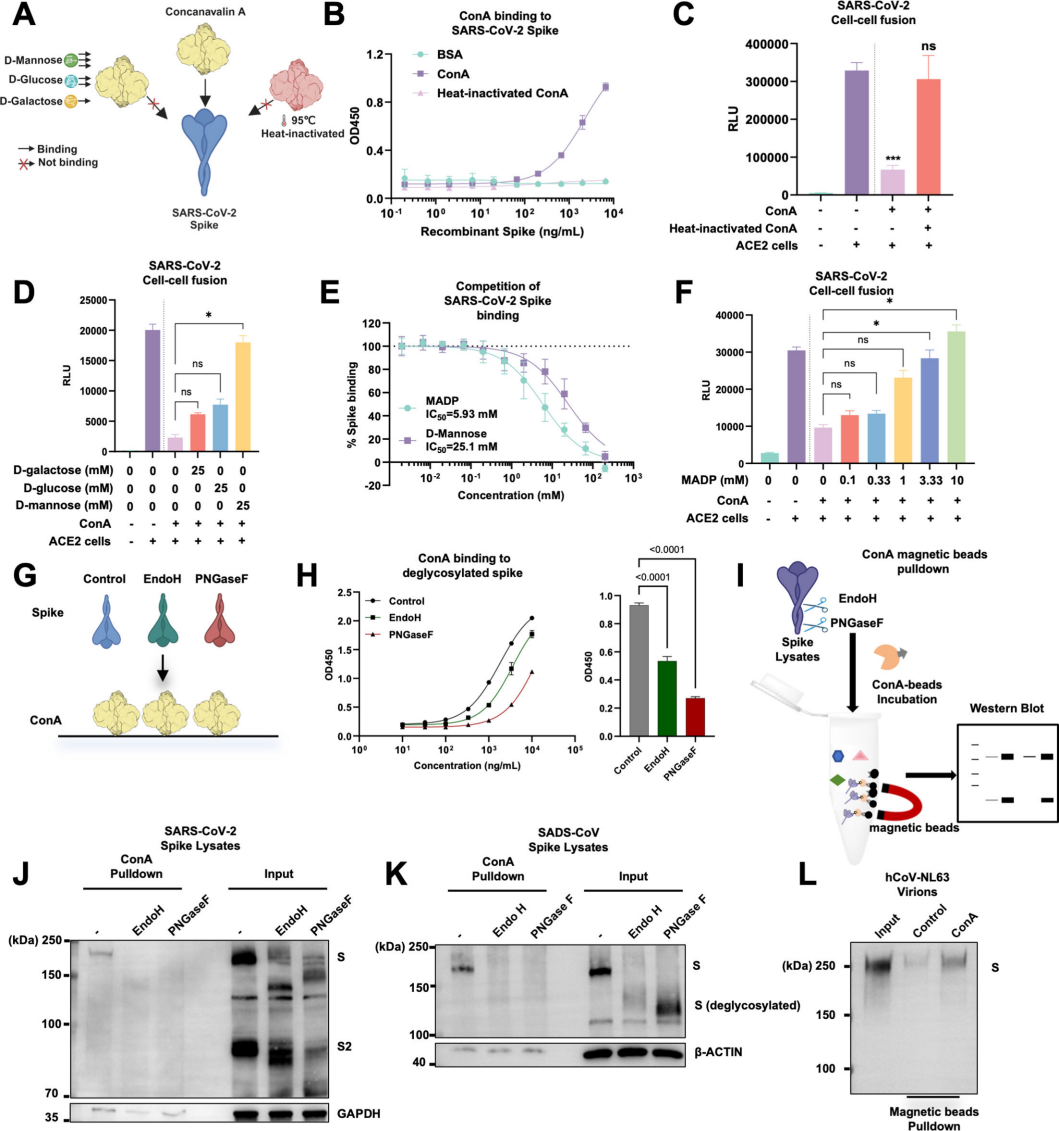
Concanavalin A directly interacts with spike via N-linked glycans. (**A**) A schematic of ConA heat inactivation and its effect on spike binding. (**B**) Indirect ELISA of His-tag containing soluble spike ectodomain binding to the immobilized native or heat-inactivated ConA. (**C**) Luciferase activity (RLU) detected from cell lysates of Cre and SARS-CoV-2 spike-expressing cells, co-cultured with LoxP-luciferase control HEK293T or HEK293T-ACE2 for 16 h in the absence or presence of 196.07 nM (20 µg/mL) native or heat-inactivated ConA. Data are representative of three individual repeats. (**D**) Luciferase activity (RLU) detected from cell lysates of Cre and SARS-CoV-2 spike-expressing cells, co-cultured with LoxP-luciferase control HEK293T or HEK293T-ACE2 for 16 h in the absence or presence of 196.07 nM native ConA and various types of monosaccharides; 25 mM D-glucose, D-mannose, and D-galactose were supplemented at the beginning of the co-culture assay. Data are representative of three individual repeats. Data are representative of three repeats, and data points are represented as mean ± SEM. Statistical significance was determined using one-way ANOVA with Sidak post-hoc multiple comparison test. *P* values are indicated as *ns,* not significant, *P < 0.05, ***P < 0.0005. (**E**) Competition ELISA of 500 ng/mL soluble spike ectodomain binding to the immobilized native ConA in the presence of increasing concentrations (0.002–200 mM) of D-mannose or Methyl alpha-D-mannopyranoside (MADP). (**F**) Luciferase activity (RLU) was detected from cell lysates of Cre and SARS-CoV-2 spike-expressing cells, co-cultured with LoxP-luciferase control HEK293T or HEK293T-ACE2 for 16 h in the absence or presence of 196.07 nM native ConA and various concentrations of methyl alpha-D-mannopyranoside (MADP); 0.1–10 mM MADP was supplemented at the beginning of the co-culture assay. Data are representative of three individual repeats. (**G**) Schematics of ConA ELISA with purified prefusion spike trimers treated with Endo H- and PNGase F. (**H**) ConA ELISA showing the binding of control, Endo H-, or PNGase F-treated prefusion spike HexaPro ectodomain (S-6P-ECD) to the ELISA plate coated with ConA; relative binding of ConA to untreated and treated spikes at 1 μg/mL was shown in the bar chart. (**I**) Schematics of ConA-mediated pulldown of spike or deglycosylated spike from cell lysates. (**J**) Immunoblots showing the full-length SARS-CoV-2 spike and S2, collected from the ConA magnetic beads pulldown from control, Endo H- or PNGase F-treated cell lysate samples. Total cell lysates were used as input controls. Blots are representative of three individual repeats. (**K**) Immunoblots showing the full-length SADS-CoV spike and S2, collected from the ConA magnetic beads pulldown from control, Endo H- or PNGase F-treated cell lysate samples. Total cell lysates were used as input controls. Blots are representative of two individual repeats. (**L**) Immunoblots showing the full-length hCoV-NL63 spike collected from the ConA magnetic beads pulldown from hCoV-NL63 viral supernatants. Viral supernatants were used as input controls, and protein A/G magnetic beads were used as negative controls. Blots are representative of three individual repeats.

Since ConA is a legume lectin, we investigated whether ConA physically engages the spike through its carbohydrate-binding capacity. To elucidate its inhibitory effects on spike-mediated cell-cell fusion, a series of biochemical assays was conducted to investigate the binding mode of ConA to the spike. First, we conducted heat inactivation of ConA to examine whether carbohydrate-binding activity is essential for its interaction with the SARS-CoV-2 spike protein (Fig. 3A). After treatment with ConA at 95°C for 30 min, we found that heat-inactivated ConA immobilized on a plate completely lost its binding capacity to the prefusion spike ectodomain compared to the native ConA (Fig. 3B), as measured by an indirect ELISA assay. When the same batch of heat-inactivated ConA was used in a cell-cell fusion assay, the loss in spike binding was directly translated into the significant loss of ConA inhibitory activity against the SARS-CoV-2 spike-mediated membrane fusion with HEK293T-ACE2 cells when compared to the native ConA (Fig. 3C). These data suggested that ConA-mediated spike inhibition requires its carbohydrate-binding property.

To understand the specificity of N-linked glycosylation modification required for ConA binding, we examined whether supplementing monosaccharides, such as D-glucose, D-mannose, or D-galactose, could compete for the ConA-binding pocket and interfere with its inhibition of the spike (Fig. 3C). When 25 mM of D-mannose, D-galactose, or D-glucose was supplemented in supernatants of the cell-cell fusion assay, only D-mannose antagonized the inhibitory effect of ConA on SARS-CoV-2 spike-mediated cell-cell fusion (Fig. 3D), suggesting that the mannose binding property of ConA is required for the inhibition of spike-mediated membrane fusion. To confirm that the mannose-binding properties of ConA contribute to the inhibition of spike-mediated membrane fusion, we also tested methyl-α-D-mannopyranoside (MADP), a mannose derivative that binds to the ConA-binding pocket with stronger affinity. Similar to D-mannose, MADP also dose-dependently antagonized the binding of ConA to the spike (Fig. 3E), as well as restored the spike-mediated membrane fusion with ACE2-expressing cells in the presence of ConA (Fig. 3F). These data suggested that ConA recognizes the mannose sugar moiety displayed on the SARS-CoV-2 spike trimer and impedes spike-mediated membrane fusion.

Since ConA-mediated recognition of mannose sugars displayed on spike N-linked glycosylation is essential, we tested whether the removal of carbohydrates from the spike trimer would abolish its binding to ConA. When we pretreated spike-expressing cell lysates with recombinant endoglycosidase H (EndoH) or peptide N-glycosidase F (PNGase F), we probed whether the removal of high-mannose oligosaccharides or all N-linked glycans is necessary to reduce the interaction between the spike protein and ConA (Fig. 3G). After validating the deglycosylation of spike by these glycosidases (Fig. S3A), the ELISA binding assay demonstrated that ConA coated on the ELISA plate showed reduced binding to either EndoH or PNGaseF-treated purified SARS-CoV-2 prefusion spikes (Fig. 3H). Moreover, we also performed affinity pulldown of spikes from cell lysate samples using ConA-coated magnetic beads (Fig. 3I). Indeed, removal of oligomannose modification from spike by EndoH was sufficient to prevent the affinity pulldown of the WT SARS-CoV-2 spike from whole cell lysates using ConA-conjugated magnetic beads (Fig. 3J). Moreover, we also tested the SADS-CoV spike, an α-coronavirus genus spike that is highly divergent from the SARS-CoV-2 spike. Similarly, EndoH treatment was sufficient to abolish the affinity pulldown of spike from ConA-conjugated magnetic beads (Fig. 3K). Furthermore, use of ConA and control Protein A/G magnetic beads pulled down viral supernatants containing authentic hCoV-NL63 demonstrated a direct interaction of ConA with spikes present on the hCoV-NL63 viral particles (Fig. 3L). These data suggested that ConA physically interacts with N-linked glycosylation, displaying mannose sugars on the coronavirus spike proteins.

### Concanavalin A binding to conserved mannose glycans interferes with coronavirus spike proteolytic processing

**Fig 4:**
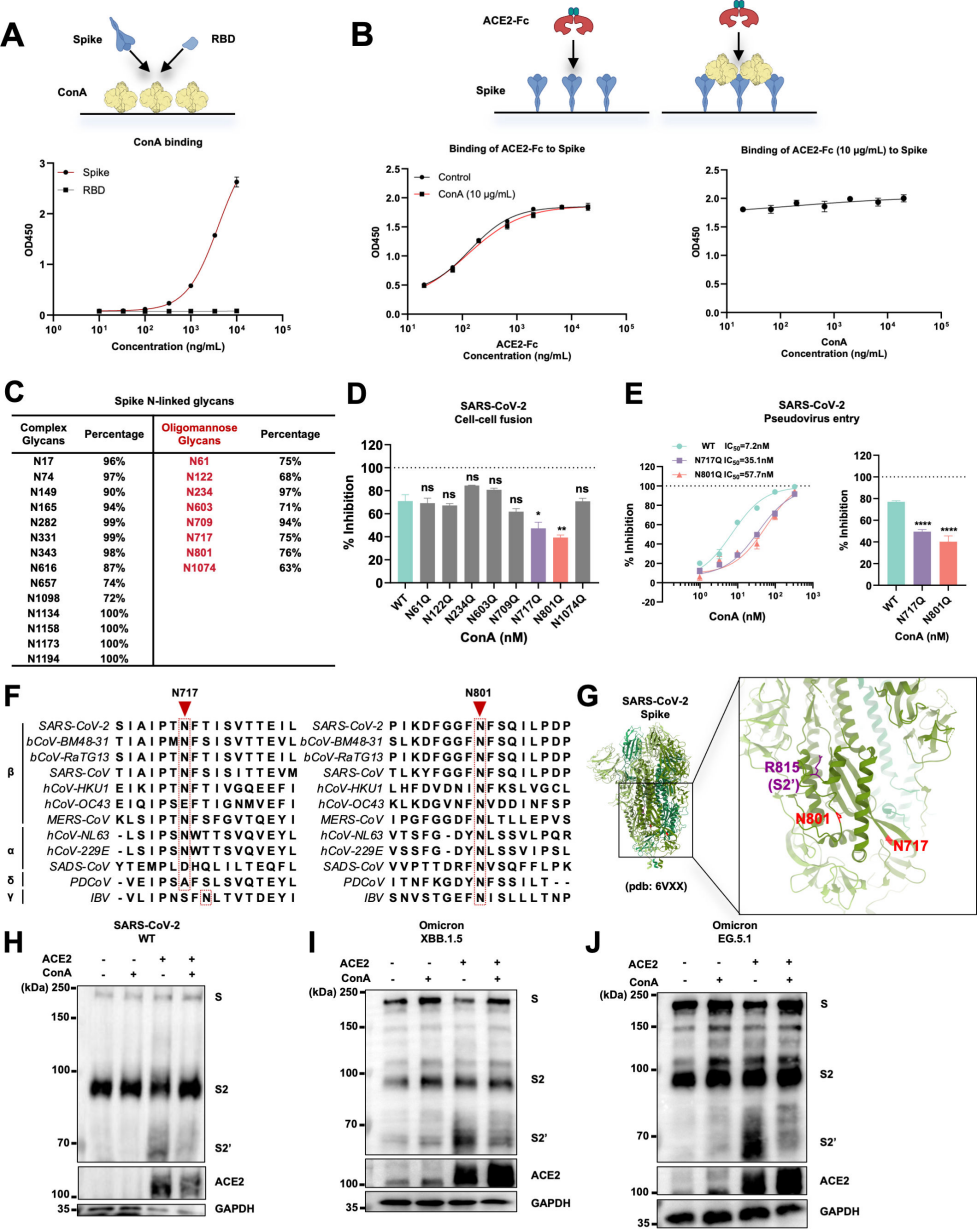
Concanavalin A binding to conserved mannose glycans interferes with coronavirus spike proteolytic processing. (**A**) Indirect ELISA showing immobilized ConA binding to 1 ng/mL to 10 μg/mL half-logarithmic dilutions of prefusion spike HexaPro ectodomain (S-6P-ECD), but not the same concentrations of the receptor binding domain (RBD). Data are representative of two replicates. (**B**) Indirect ELISA showing immobilized S-6P-ECD binding to half-logarithmic dilutions of 3.33 ng to 33.33 μg/mL soluble ACE2-Fc in the absence or presence of 10 μg/mL ConA (left) or half-logarithmic dilutions of 3.33 ng to 33.33 μg/mL ConA to 10 μg/mL ACE2-Fc (right). Data are representative of two replicates. (**C**) A table summarizing the reported percentage of oligomannose and complex sugar occupancies on the SARS-CoV-2 WT spike protein. (**D**) ConA-mediated inhibition of spike-mediated cell-cell fusion, derived from cells co-expressing 8 individual glycosylation mutant spikes (N61Q, N122Q, N234Q, N603Q, N709Q, N717Q, N801Q, and N1074Q) and Cre, co-cultured with LoxP-luciferase HEK293T-ACE2 cells for 16 h. Percentage inhibition was normalized against the bioluminescence readings from each spike mutant in the absence of ConA. The concentration of ConA used was 196.07 nM (20 µg/mL). Data are representative of four repeats, and data points are represented as mean ± SEM. (**E**) The IC_50_ of ConA on pseudoviruses bearing the WT BA.4, N717Q, and N801Q spike mutants in HEK293T-ACE2 cells. The inhibitory activity of ConA at 32.64 nM (3.33 µg/mL) on pseudovirus entry mediated by the WT, N717Q, and N801Q spike mutants was shown as a bar chart. Data are representative of four repeats, and data points are represented as mean ± SEM. Statistical significance was determined using one-way ANOVA with Sidak post-hoc multiple comparison test. *P*-values are indicated as *ns,* not significant, *P < 0.05, **P < 0.005, ****P < 0.0001. (**F**) Amino acid sequence alignment of spikes N717 and N801 regions with reference to the SARS-CoV-2 spike. (**G**) Structural representation of SARS-CoV-2 spike N717 and N801 residues modeled on the prefusion spike (pdb: 6VXX). (**H**) Immunoblots of full-length spike, S2, and cleaved S2′ collected from SARS-CoV-2 WT spike-expressing HEK293T cells co-cultured with control HEK293T or HEK293T-ACE2 cells in the absence or presence of 196.07 nM ConA. Blots are representative of two repeats. (**I**) Immunoblots of full-length spike, S2, and cleaved S2′ collected from SARS-CoV-2 Omicron XBB.1.5 spike-expressing HEK293T cells co-cultured with control HEK293T or HEK293T-ACE2 cells in the absence or presence of 196.07 nM ConA. Blots are representative of two repeats. (**J**) Immunoblots of full-length spike, S2, and cleaved S2′ collected from SARS-CoV-2 Omicron EG.5.1 spike-expressing HEK293T cells co-cultured with control HEK293T or HEK293T-ACE2 cells in the absence or presence of 196.07 nM ConA. Blots are representative of two repeats.

To further elucidate the mode of action of ConA on spike, we investigated the binding of purified SARS-CoV-2 prefusion spike ectodomain or the receptor binding domain (RBD) with immobilized ConA-coated ELISA plates. Interestingly, we observed dose-dependent binding of spike to the immobilized ConA, but minimal binding of RBD up to 10 μg/mL concentration (Fig. 4A). This suggested that ConA-mediated inhibition of spike could be independent of spike-receptor interaction. To validate this result, we performed a reciprocal binding assay in which the full-length spike was immobilized and incubated with recombinant ACE2-Fc in the absence or presence of ConA. The presence of 10 μg/mL ConA did not reduce ACE2-Fc binding to the immobilized spike at various concentrations tested (Fig. 4B, left); similarly, ConA concentrations up to 326.5 nM (33.3 μg/mL) had no effect on the binding of 10 μg/mL ACE2-Fc to immobilized spike (Fig. 4B, right). These results demonstrate that ConA does not inhibit spike and ACE2 interaction.

To functionally rule out the possibility that the inhibitory effect of ConA results from interfering with the interaction between the receptor binding motif (RBM) and ACE2, we employed a recently published customized viral receptor, Nb30-CVR (14). Nb30 is a camelid-derived nanobody that recognizes the Class IV epitope of spike RBD, which is distal from the ACE2-interacting RBM (15); binding of Nb30-CVR to this epitope permits SARS-CoV-2 pseudovirus entry and cell-cell fusion independent of ACE2(14). Our results showed that ConA also dose-dependently inhibited spike-mediated cell-cell fusion with either Nb30-CVR cells or ACE2 cells at a similar dose range (Fig. S3B). Together, these results indicate that ConA does not compete with ACE2 for spike binding.

Since the effect of ConA requires mannose glycans displayed on the spike protein and is independent of RBD binding, we individually tested 8 of 22 N-glycans on the spike protomer that were previously reported to be decorated with high occupancies of oligomannosidic modifications (13) (Fig. 4C). In line with our spike and RBD binding assay, 8 residues reported to be occupied by 8 oligomannosidic modifications were all outside the RBD region. By using site-directed mutagenesis to substitute each oligomannosidic N-linked glycosylation sites to glutamine (Q), we found that only N717Q and N801Q substitutions within the S2 subunit significantly reduced the ConA-mediated inhibition of cell-cell fusion to less than 50% at 196.06 nM (Fig. 4D). Furthermore, we also prepared pseudovirus particles carrying the individual N717Q and N801Q spike substitutions. When compared to the WT spike, N717Q or N801Q substitutions significantly increased the IC_50_ of ConA on pseudovirus entry from 7.15 nM to 35.05 nM and 57.73 nM, respectively (Fig. 4E, Left). At a concentration of 32.64 nM, ConA exhibited significantly less inhibition on pseudovirus entry mediated by the spike N717Q or N801Q mutants (Fig. 4E, Right). These data suggested that the oligomannosidic glycans at N717 and N801 residues of the SARS-CoV-2 spike contribute to the ConA binding and are mainly responsible for ConA-mediated inhibition of spike.

By performing an amino acid sequence alignment of other coronavirus spikes, we found that the N-glycosylation modification of the SARS-CoV-2 spike N801 residue is highly conserved in all coronavirus spikes listed (Fig. 4F); comparably, the N717 residue is also highly conserved, with exceptions in the hCoV-OC43, SADS-CoV, and PDCoV spikes (Fig. 4F). Structural modeling of SARS-CoV-2 ancestral spike trimer based on the published Cryo-electron microscopy (pdb: 6VXX) suggested that these two N-glycans were both located proximally to the putative S2′ cleavage site residue R815 (Fig. 4G); similarly for the SADS-CoV spike protein (pdb: 6M16), the residue N654, which is equivalent to SARS-CoV-2 N801, is also located adjacent to the S2′ cleavage site residue R673 (Fig. S3C). Hence, the interaction of ConA with spike on these two residues could affect the proteolytic event required for spike-mediated membrane fusion.

To investigate the potential effect of ConA on spike proteolytic activation, we performed the cell-cell fusion assays using SARS-CoV-2 WT, XBB.1.5, and EG.5.1 spikes in the absence or presence of 196.06 nM ConA and co-cultured with control or ACE2-expressing HEK293T cells for 24 h and immunoblotted the cell lysates containing potentially cleaved spike species. In the absence of ACE2, addition of ConA had no effect on the full-length S and cleaved S2 at 190 kDa and 98 kDa, respectively (Fig. 4H through J). In the presence of ACE2-expressing cells, spike is readily processed into the S2′ species at 68 kDa in cells expressing the WT, XBB.1.5, and E.G.5.1 variants (Fig. 4H through J, lane 3). Addition of ConA had no effect on the full-length spike or cleaved S2 but significantly reduced the cleaved S2′ species not only in the WT spike (Fig. 4H, lane 4) but also in co-cultured cell lysates from Omicron XBB.1.5 (Fig. 4I, lane 4) and EG.5.1 spike variants (Fig. 4J, lane 4). These data suggested that ConA interacts with spikes on the conserved N-linked glycosylation sites at N717 and N801 residues and inhibits SARS-CoV-2 spike proteolytic activation and subsequent membrane fusion.

### Broad-spectrum inhibition of authentic coronavirus entry *in vitro*

**Fig 5:**
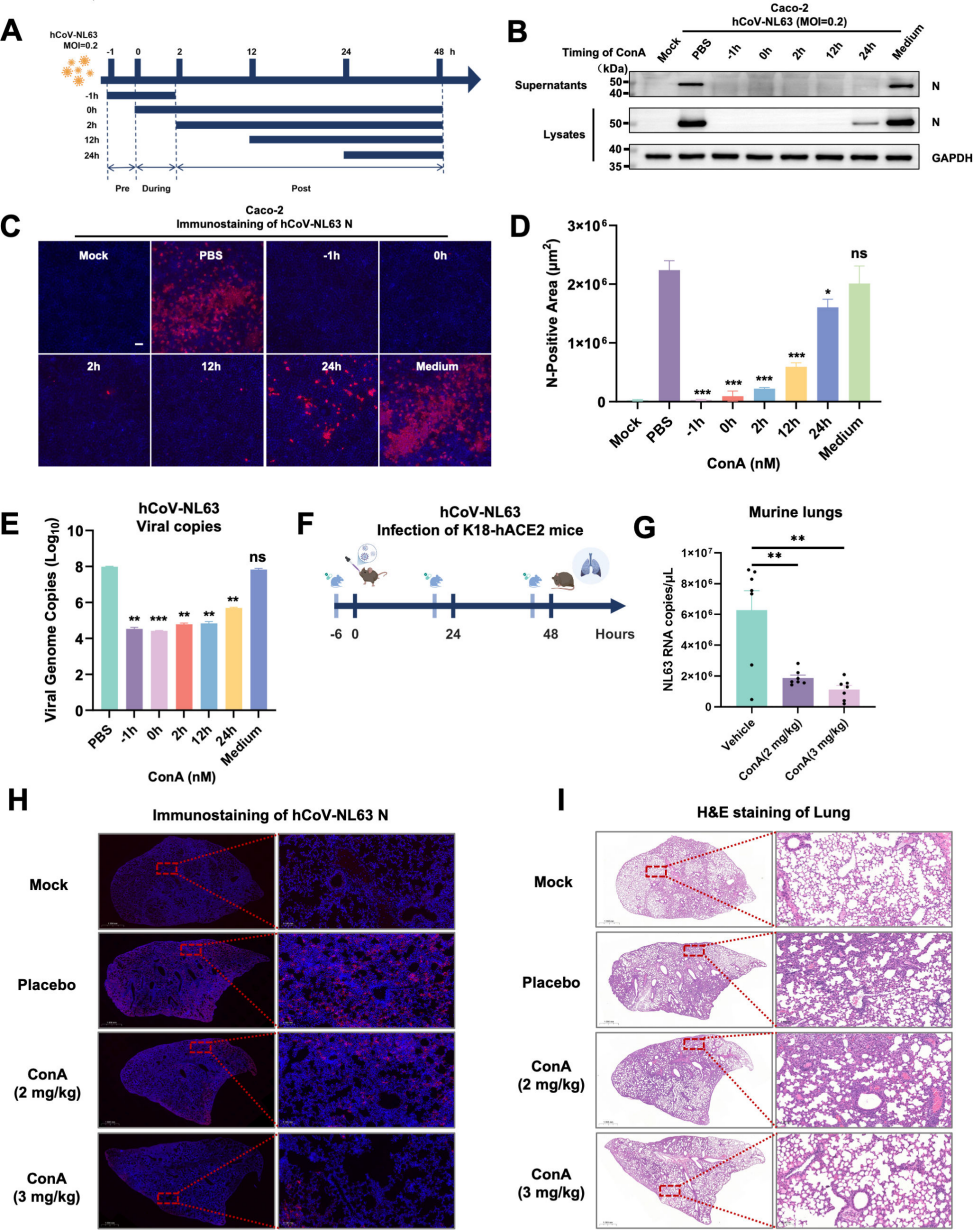
Broad-spectrum inhibition of authentic coronavirus infections *in vitro* and *in vivo.* (**A**) The schematic of the hCoV-NL63 infection model with ConA added at various time points on Caco-2 cells prior or after infection. (**B**) Immunoblots of hCoV-NL63 nucleocapsid (N), detected from supernatants and lysates, where Caco-2 cells were inoculated with (MOI = 0.2) hCoV-NL63 for 48 h in the presence of 196.07 nM (20 µg/mL) ConA added at indicated time points. (**C**) Representative fluorescent images of hCoV-NL63 N and DAPI counterstain in the 4% paraformaldehyde fixed Caco-2 cells, where Caco-2 cells were inoculated with MOI = 0.2 hCoV-NL63 in the absence or presence of 196.07 nM ConA added at indicated time points. The scale bar is representative of 100 μm. (**D**) Quantified cyanine 3 (Cy3) fluorescence area (μm^2^) per field of view based on hCoV-NL63 N immunostaining described in (C). (**E**) Viral RNA copies level of hCoV-NL63 N genes in the 48-h infectious supernatants of the Caco-2 cells were quantified by quantitative PCR. (**F**) A schematic of the ConA pretreatment in a murine model of hCoV-NL63 infection. PBS control and ConA prepared in PBS were administered intranasally 6 h prior to hCoV-NL63 infection. (**G**) Viral copies of N detected from right lung whole tissue homogenates of mice infected with 1.5 × 10^7^ FFU of hCoV-NL63, pretreated with PBS and 2 or 3 mg/kg ConA. Each data point represents an individual mouse, *n* = 7. Data points are represented as mean ± SEM. Statistical significance was determined using one-way ANOVA with Sidak post-hoc multiple comparison. *P* values are indicated as ns*,* not significant and **P < 0.005, ***P < 0.0005. (**H**) Representative immunofluorescence images of hCoV-NL63 N protein in the lung sagittal slices derived from (G). N was fluorescently labeled with Cy3, and the background was counterstained with DAPI. Scale bars are indicative of 1000 μm and 100 μm, respectively. (**I**) Representative hematoxylin and eosin (H&E) staining of murine lung sagittal slices derived from (G). Scale bars are indicative of 1000 μm and 100 μm, respectively.

To fully elucidate the kinetics of ConA on replicating coronaviruses, we employed various time points to study the effect of ConA on endemic coronavirus hCoV-NL63 coronavirus infections (Fig. 5A). For 1 h prior to or during the 2-h inoculation period on Caco-2 cells, the addition of ConA completely abrogated the initial hCoV-NL63 infection. This was evidenced by the absence of nucleocapsid (N) protein detection in both supernatants and cell lysates of Caco-2 cells 48 h post-infection (h.p.i.), compared to untreated controls. Notably, the addition of ConA 12 h after the initial infection still resulted in a significant reduction in the hCoV-NL63 infection; however, this inhibitory effect was lost when ConA was added 24 h post-infection (Fig. 5B). These findings highlight the critical role of ConA-mediated inhibition during the early stages of viral entry and replication. ConA-mediated inhibition of hCoV-NL63 was further validated by immunostaining, where ConA markedly decreased the amount of N stained by CY3-conjugated secondary antibodies per field of view (Fig. 5C). Furthermore, quantitative PCR analysis of viral RNA levels of N genes in infectious supernatants mirrored the inhibitory effects of ConA across different time points (Fig. 5D). Hence, these findings highlight that ConA-mediated inhibition predominantly acts during the viral entry step.

Since the staining of hCoV-NL63 provides an accurate estimation of viral entry into Caco-2 cells, we performed a half-logarithmic dilution of ConA and quantified its dose-response inhibition based on immunostaining of N (Fig. S4A). The IC_50_ of ConA for authentic hCoV-NL63 infection in Caco-2 cells was determined to be 9.96 nM (1.01 µg/mL) (Fig. S4A) and is similar to the IC_50_ of ConA for SARS-CoV-2 WT infection on Caco-2 cells (Fig. 1E). These results collectively underscore that ConA-mediated inhibition of hCoV-NL63 primarily occurs during the viral entry step, as pretreatment with ConA efficiently blocks α-coronavirus hCoV-NL63 entry into Caco-2 cells. To further validate the broad-spectrum inhibitory activity against α- and β-coronaviruses infections, we also tested the recombinant hCoV-OC43 expressing a GFP reporter (hCoV-OC43-GFP) and recombinant SADS-CoV-DsRed. Pre-incubation of ConA 1 h before or during the infection potently inhibited the hCoV-OC43-GFP and SADS-CoV-DsRed fluorescence signals in Caco-2 cells (Fig. S4B and C). These data confirmed that ConA also exhibited a broad-spectrum inhibitory effect against authentic coronavirus infections.

### Intranasal delivery of ConA inhibits hCoV-NL63 coronavirus infection *in vivo*

Before evaluating the effects of ConA on authentic viral infections *in vivo*, we assessed its potential toxicity following intranasal delivery in mice. This preliminary analysis was crucial to ensure that any observed effects of ConA on viral infection were not confounded by host toxicity or adverse physiological responses. ConA dissolved in phosphate buffer saline (PBS) was prepared in 50 μL and intranasally instilled at 1.5, 3, and 4.5 mg/kg concentrations in mice. Four days after ConA treatment, no significant reduction in body weight was observed in mice intranasally delivered with ConA or the PBS control (Fig. S5A). The same mice were sacrificed for histological assessment of ConA treatment in the lungs and livers. Instillation of ConA alone into the mouse respiratory tract also did not result in the immune cell infiltration into the lung or liver (Fig. S5B and C), validated by the hematoxylin and eosin (H&E) staining. Moreover, intranasally administered ConA at 3 mg/kg did not cause long-term pathological outcomes, since no body weight reduction and pathological abnormality in mice was observed even 7 and 10 days of ConA treatment when compared to the PBS control mice (Fig. S5D and E). These data suggested that intranasal delivery of ConA at a dose between 1.5 and 4.5 mg/kg is safe for the subsequent analysis of viral infections.

To assess the antiviral effect of ConA on hCoV-NL63 infection *in vivo*, we utilized K18-hACE2 transgenic mice expressing human ACE2 under the control of the K18 promoter. Each mouse was intranasally administered PBS (control) or 2 or 3 mg/kg ConA 6 h prior to intranasal inoculation with 1.5 × 10^7^ FFU of hCoV-NL63 and once daily after the infection (Fig. 5F). The results showed that ConA treatment doses significantly reduced viral N gene copies in RNA extracted from infected lungs (Fig. 5G). This reduction in infection was confirmed by immunofluorescence staining, where ConA significantly decreased the area of N protein-positive cells in the sagittal lung sections (Fig. 5H). Furthermore, hematoxylin and eosin (H&E) staining of matched lung sections revealed reduced immune cell infiltration and inflammation in ConA-treated mice when compared to the control mice (Fig. 5I). These data demonstrate that ConA treatment effectively reduces hCoV-NL63 infection and associated pathology *in vivo*.

## DISCUSSION

The oligomannosidic modification of viral glycoproteins represents a unique form of N-linked glycosylation that is distinct from the glycosylation patterns typically observed on host cell membrane proteins. This distinct glycan profile provides an exploitable vulnerability for antiviral strategies, such as legume lectin-based inhibitors like ConA. Our study establishes ConA as a broad-spectrum coronavirus entry inhibitor through a three-tiered validation framework: spike-mediated cell-cell fusion assays, pseudoviral entry models, and authentic virus challenge experiments *in vitro* and *in vivo*. Mechanistically, the anti-spike activity of ConA is mediated through its unique mannose-binding specificity, as demonstrated by the competitive inhibition assays with D-mannose and structural analogs of MADP. Importantly, the binding of ConA to oligomannosidic glycan directly interferes with the spike-mediated membrane fusion at both viral entry (virus-cell) and syncytia formation (cell-cell) interfaces. This conserved glycan signature across coronavirus spikes suggests that the antiviral activity of ConA may extend broadly to other members of the coronavirus spikes, as demonstrated by the similar antiviral activity of ConA against authentic SARS-CoV-2 and hCoV-NL63 infections *in vitro*. Compared to the highly pathogenic coronaviruses, the hCoV-NL63 virus is less virulent and causes milder disease in mice (36). In all cases, hCoV-NL63 infection did not cause mortality or weight loss in the mice but was sufficient to evaluate the effectiveness of viral inhibitors such as AL5E or host interferon responses (36–38). Importantly, our mouse model of hCoV-NL63 infection has demonstrated a comparable level of viral replication, while ConA treatment effectively reduced viral loads in the murine lungs 2 days after infection. These findings not only highlight the potential of lectin-based therapeutics for combating current and emerging coronaviruses but also underscore the importance of glycan-targeting strategies in antiviral research (39).

While the SARS-CoV-2 spikes constantly evolve against neutralizing antibodies, our study identified two conserved N-linked glycosylation sites (N717 and N801) within the spike S2 subunit as critical vulnerability hotspots for spike-mediated membrane fusion. Notably, the SARS-CoV-2 RBD contains only two complex-type glycosylation sites and lacks oligomannosidic modifications required for ConA binding (Fig. 4A). Moreover, ConA did not compete with ACE2 on spike binding, suggesting that ConA-mediated inhibition of spike is receptor-independent. Despite the widespread mutational evolution observed in the S1 subunit (40), the oligomannosidic glycosylation motifs at spike N717 and N801 remain highly conserved across SARS-CoV-2 variants and phylogenetically related coronavirus strains. Functional analyses of ConA on spike-mediated cell-cell fusion and pseudovirus entry models established that ConA-mediated antiviral activity primarily depends on its binding to oligomannosylated N717/N801 residues (Fig. 4D). This interaction likely achieves the inhibition through three non-exclusive mechanisms: (i) steric obstruction of S2′ proteolytic cleavage, (ii) reduced accessibility for host proteases (e.g., TMPRSS2 or cathepsins), and (iii) prevention of post-priming structural rearrangements in the S2 subunit. Unlike the viral glycan shield that primarily evades host adaptive immune responses (13, 15), the converging N-linked glycosylation sites at N717 and N801 could be important for the folding of spikes during their maturation. Therefore, these glycans are exceptionally conserved across spikes from other coronavirus genera, including hCoV-NL63, MERS-CoV, and SADS-CoV.

During the preparation of this manuscript, a concurrent study demonstrated that ConA can induce the physical aggregation of SARS-CoV-2 viral particles, resulting in a loss of infectivity by inactivating virions (29). In line with this report, we performed negative-staining transmission electron microscopy and observed large aggregates between ConA and spike when these proteins were mixed in various fields of the electron micrographs (Fig. S3D), but not ConA or spike alone (Fig. S3E). As a result, further structural classification and analysis of spike and ConA binding are unavailable. We think that ConA and spike trimers may crosslink and aggregate viral particles and exert direct virucidal effect as an important mode of action previously proposed (29). Taken together, we propose that there are two inhibitory mechanisms of the ConA: First, ConA induces the aggregation of viral particles prior to cell attachment or viral entry, thereby reducing the viral infectivity. It was originally proposed by Klevanski et al. and reconfirmed by our negative-staining transmission EM. Second, ConA can directly inhibit the S2′ proteolytic processing during viral entry or syncytia formation, and it can be achieved via binding to the N717 and N801 without affecting ACE2 binding or virion aggregation. These findings highlighted the potential of targeting the conserved N717/N801 glycosylation sites for developing broad-spectrum, lectin-based antivirals against coronaviruses.

Plant-derived lectins represent a promising class of antiviral agents due to their natural abundance, scalability for production, and inherent stability in biological environments. In murine models of respiratory virus infections, exogenous plant-derived lectins, such as engineered banana lectin (H84T) (24), lectin from hyacinth beans and griffithsin (41, 42), have demonstrated mucosal compatibility and can offer protection against viral infections such as influenza and SARS-CoV-2 *in vivo*. Although our work primarily focuses on the antiviral effect of ConA on coronavirus cellular entry, further work on its compatibility and half-life in the respiratory tract *in vivo* is necessary. The timing of ConA administration warrants further investigation (43), as our data mainly demonstrated its prophylactic effect against the hCoV-NL63 infection. In contrast to neutralizing antibodies and host adaptive immune responses, the use of ConA may serve as an early intervention to reduce viral load during the acute infection phase. A study has demonstrated that conjugating a lectin with EK1 (a broad-spectrum coronavirus peptide inhibitor) increases antiviral efficacy by more than 20-fold compared with the lectin alone (42); it is also possible to improve the ConA’s antiviral potential by conjugating it with EK1 or other fusion inhibitors. Thus, exogenous lectins could serve as a first-line defense by targeting conserved viral entry checkpoints, which would circumvent both the antibody evasion seen in the SARS-CoV-2 variants and the potential risks associated with late-phase, antibody-dependent antiviral approaches.

## MATERIALS AND METHODS

### Viruses and biosafety

The human coronavirus hCoV-NL63 NR-470 strain was obtained from Prof. Jincun Zhao and cultured in Caco-2 cells at 34°C (38). The SADS-CoV-DsRed strain was obtained from Prof. Yanhua Li (Yangzhou University) and cultured in Huh-7-TMPRSS2 cells (44). Cell experiments involving authentic hCoV-NL63 and SADS-CoV virus infection were conducted in a biosafety level 2 (BSL-2) laboratory, while murine infection of hCoV-NL63 was performed at the animal biosafety level 2 (ABSL-2) facility of the Guangzhou Laboratory animal center.

For ConA inhibition of authentic SARS-CoV-2 infection on Caco-2 cells.

### Research animal and murine infection model of hCoV-NL63

Female H11-K18-hACE2 and C57BL/6 mice aged 6–8 weeks were used in this study. Specific pathogen-free (SPF) female H11-K18-hACE2 mice were maintained under SPF conditions at the Guangzhou Laboratory animal center in compliance with regulations in the Guide for the Care and Use of Laboratory Animals. The experimental protocol used in this project was approved by the Guangzhou Laboratory ethics committee (permission no. GZLAB-AUCP-2024-11-A05).

For *in vivo* toxicity experiments, C57BL/6 mice were administered intranasally with vehicle (PBS) or ConA at 1.5, 3.0, and 4.5 mg/kg. Weight changes were monitored and recorded for 4 days after treatments. Later, mice lungs and livers were harvested for Hematoxylin and Eosin (H&E) staining. For ConA treatments, a 2.0 or 3.0 mg/kg dose of ConA or vehicle only (PBS) was administered intranasally 6 h prior to authentic infection and once daily for 2 days. Subsequently, H11-K18-hACE2 mice were inoculated intranasally with 70 μL of virus dilution containing 1.5 × 10^7^ FFU of NL63 virus. Mice lungs were collected on day 2 post-infection, and the number of viral genome copies was quantified using qPCR. Histopathological changes and viral infection status in lung tissues were evaluated by H&E staining and immunohistochemical analysis of the viral N protein, respectively.

### Tissue culture and generation of stable cell lines

HEK293T, A549, and Caco-2 cells were cultured in Gibco Dulbecco’s modified Eagle’s medium (DMEM) (Gibco, C11995500BT) supplemented with 10% fetal bovine serum (FBS) (Excell, FAP500) and 1% penicillin/streptomycin (P/S) (Invitrogen,15140122) at 37°C with 5% CO_2_ in a humidified incubator. All cell lines were routinely tested for mycoplasma contamination by PCR; passages between 3 and 20 were used. HEK293T cells over-expressing human ACE2 (HEK293T-ACE2) and Huh-7-ACE2 were generated using a lentivirus, prepared using the pLVX-ACE2, psPAX2, and pMD.2 plasmids at a 2:3:1 ratio, respectively; 48 h post-transduction, HEK293T-ACE2 were selected for antibiotic resistance using 2 μg/mL puromycin and maintained for 1 week before use. Huh-7 cells over-expressing the TMPRSS2 (Huh7-TMPRSS2) were generated using a lentivirus, prepared using the pLVX-TMPRSS2 derived from a published study (11); 48 h post-transduction, Huh7-TMPRSS2 were selected for antibiotic resistance using 2 μg/mL puromycin and maintained for 1 week.

### Chemical reagents and antibodies

Concanavalin A used in this study was commercially purchased from Sigma-Aldrich (C0412-5MG) and was dissolved in PBS. Control lectins such as peanut agglutinin (PNA) (B-1075-5) and wheat germ agglutinin (WGA) (RL-1022-5) were purchased from Vector Laboratory. Recombinant glycosidase Endo H (P0702L) and PNGase F (P0704L) were purchased from New England Biolabs and were used according to the manufacturer’s instructions prior to pulldown assays. For the ConA-mediated pulldown of spike protein, BeyoMag concanavalin A magnetic beads (P2156-1mL) were purchased from Beyotime. D-Glucose (G7528-1KG) was purchased from Sigma-Aldrich, whereas Methyl-α-D-mannopyranoside (MADP, M813663-100g) and D-Mannose (D813082-25g) were purchased from Macklin. D-Galactose (A600215-0025) was purchased from BBI. Recombinant SARS-CoV-2 Spike S1+S2 ectodomain (S-ECD) (B.1.1.529/Omicron) trimer protein (RP02111) was purchased from Abclonal.

Anti-ACE2 Rabbit Polyclonal Antibody (21115-1-AP) was purchased from Proteintech. Rabbit anti-SARS-CoV-2 S2 (40590-T62) polyclonal antibody was used for the detection of S2′, S2, and S proteins. Rabbit anti-human coronavirus (HCoV-NL63) nucleocapsid (N) antibody (40641-T62, SinoBiological) was used for the detection of N. Mouse anti-Strep II-Tag mAb (AE066, Abclonal) was used for the detection of SADS-CoV S protein. Rabbit anti-SARS-CoV/SARS-CoV-2 N conjugated with horseradish peroxidase (HRP) antibody (40143-R001-H-20) was purchased from SinoBiological. Loading controls were blotted using the anti-GAPDH (HRP-60004) and anti-β-actin (HRP-66009) monoclonal antibodies, purchased from Proteintech. HRP-conjugated goat anti-rabbit (111-035-003) and anti-mouse (115-035-003) secondary antibodies were purchased from Jackson ImmunoResearch. Anti-His tag HRP-conjugated mouse monoclonal antibody (HRP-66005) was purchased from Proteintech and used for enzyme-linked immunosorbent assay (ELISA). 3,3′,5,5′-Tetramethylbenzidine (TMB) liquid substrate system for ELISA assay (T820901) was purchased from Macklin. For immunofluorescence, Cy3-conjugated goat anti-rabbit IgG polyclonal antibody (HA1102, HUABIO) secondary antibody was used.

### Plasmids and generation of coronavirus spike mutants

A codon-optimized gene encoding the SARS-CoV-2 wild-type (WT) spike protein (Wuhan-Hu-1; GenBank accession: QHD43419.1) was synthesized *de novo* (GenScript) and cloned into a pcDNA3.1 vector using PCR. Wild-type (WT) spike and variant constructs (Omicron BA.4, XBB.1.5) contain specific point mutations, and deletions were generated via stepwise mutagenesis. All spike constructs used for pseudovirus packaging contained a C-terminal 19-amino acid truncation (CTΔ19). Plasmids encoding the MERS-CoV spike, human betacoronavirus HKU1 (subtype A) spike, dipeptidyl peptidase-4 (DPP4), and transmembrane protease, serine 2 (TMPRSS2) were generously provided by Prof. Xiancai Ma (45). Plasmids encoding Cre recombinase and the LoxP-luciferase and LoxP-mCherry reporters were derived from previous studies (31, 46). Site-directed mutagenesis and deletions of coronavirus spikes were generated using customized primer pairs (synthesized by Sangon), KOD PLUS neo (TOYOBO) high-fidelity polymerase, and enzyme restriction digestion by Dpn I (NEB). All mutant plasmids were validated by a sequencing service provided by Sangon before use.

### Spike-mediated cell-cell fusion co-culture assays

Spike-mediated cell-cell fusion was determined based on the Cre-LoxP co-culture assay previously described (31, 46). Briefly, 2.5 × 10^5^ HEK293T cells were seeded in a 24-well plate per well the night before the transient transfection. For donor cells, plasmids encoding the SARS-CoV-2 WT, Omicron BA.4, XBB.1.5 and JN.1, MERS-CoV, SADS-CoV spikes, and Cre recombinase were packaged into Lipo8000 (Beyotime) and co-transfected into the HEK293T cells for 24 h. For recipient cells, plasmids encoding the LoxP-luciferase or LoxP-mCherry reporters were transfected into HEK293T control (without receptor expression) or HEK293T-ACE2 (receptor) cells for 24 h. Cells were subsequently gently detached using 0.25% trypsin-EDTA and pelleted at 700 × *g* for 4 min, before resuspending in DMEM containing 2% FBS and 1% P/S for co-culture assays.

The Cre-LoxP system determines cell-cell fusion by qualifying or quantifying mCherry or Luciferase reporter expression, respectively, when Cre recombinase removes the stop codon between the LoxP sites as previously described (31). Donor and recipient cells were strictly mixed at a 1:1 ratio in the 96-well plates without or with the indicated forms of ConA and incubated for 16 h at 37°C. Fluorescent images of mCherry showing syncytia formation were captured at endpoint using a 10× objective and 12-bit monochrome complementary metal oxide semiconductor (CMOS) camera installed on the CKX53 inverted microscope (Olympus). Alternatively, cells were lysed in a Nonidet P-40 lysis buffer containing 0.5% (vol/vol) Nonidet P-40, 25 mM Tris pH 7.3, and 150 mM NaCl supplemented with 1× EDTA-free PIC (TargetMol, C0001). Bioluminescence was quantified as relative luminescence unit (RLU) by mixing lysates with the Firefly Glo Luciferase reporter assay substrate (YEASEN, 11404ES80), recorded on a Synergy H1 plate reader (Biotek).

### Packaging of pseudovirus particles and pseudovirus entry

SARS-CoV-2 firefly luciferase pseudovirus particles were generated as previously described (11). HEK293T cells were seeded at 80% density overnight in T25 flasks coated with poly-D-lysine, before being transfected with 10 μg/mL plasmids encoding the SARS-CoV-2 S WT, XBB.1.5, EG.5.1 variants, or BA.4, N717Q, N801Q spike mutants, or the hCoV-HKU1A spike. One day after transfection, cells were infected with VSV (G*ΔG-luciferase) for 1 h and rinsed three times with PBS before incubating for an additional 24 h in complete DMEM at 37°C. The culture supernatants were clarified to remove cell debris and stored at −80°C.

For pseudovirus infection assays, HEK293T-ACE2 and Caco-2 cells were seeded in DMEM supplemented with 10% FBS and seeded into flat-bottom 96-well plates at a density of 2 × 10^4^ cells per well, respectively. The next day, the pseudovirus was mixed with indicated concentrations of ConA in 37°C for 1 h; cells were then infected with 50 μL of pseudovirus and incubated for 24 h at 37°C. Cells were eventually lysed in the NP40 lysis buffer, before being mixed with the Firefly Glo Luciferase reporter assay substrate for bioluminescence.

### ELISA assays for ConA and spike binding

For spike binding to lectin-coated plates, flat-bottom 96-well plates (BioJetFil) were coated with BSA, Peanut agglutinin (PNA), ConA, or heat-inactivated ConA diluted in PBS containing 500 μM Mn^2+^ and 1 mM Ca^2+^ at 1 μg per well overnight at 4°C. The plates were rinsed three times with ConA wash buffer (PBS supplemented with 0.1% Tween-20 [PBST], 500 μM Mn^2+^, and 1 mM Ca^2+^), blocked with blocking buffer (1% bovine serum albumin in DMEM) for 1 h at room temperature. Subsequently, half-logarithmic dilutions of his-tag purified spike HexaPro ectodomains (S-6P-ECD) or receptor binding domain (RBD) were added. For the carbohydrate competition assay, indicated concentrations of Methyl-α-D-mannopyranoside (MADP) and D-mannose were added in the blocking buffer and incubated with the spike for 2 h at room temperature. Plates were rinsed five times with ConA wash buffer before the horse Anti-His secondary antibody was added and incubated for 1 h at room temperature. Spike was detected using His-HRP diluted in blocking buffer at 1:2,500. Plates were eventually washed (five times with PBS/0.1% Tween-20, 500 μM Mn^2+^, and 1 mM Ca^2+^) and developed with TMB substrate (T820901, Macklin) and read at 450 nm on a plate reader (Biotek).

### Magnetic pulldown of coronavirus spike proteins

For ConA magnetic pulldown of SARS-CoV-2, hCoV-NL63, and SADS-CoV spikes, HEK293T cells transiently transfected with respective spikes were lysed in the NP-40 lysis buffer supplemented with the 1× EDTA-free protease inhibitor cocktail (TargetMol, C0001). After removing cell nuclei, input cell lysates were collected prior to treatment without or with 10 U/mL Endo H or PNGase F at 37°C for 2 h, respectively. Subsequently, the reaction was terminated on ice, and ConA magnetic beads (Beyotime, P2156, 1 mL) were added and incubated on a rotator overnight at 4°C. For experimental control, protein A/G magnetic beads (B23202, Bimake) were used as negative controls. Pull-down samples on the magnetic beads were washed three times in ConA wash buffer (NP-40 lysis buffer supplemented with 500 μM Mn^2+^ and 1 mM Ca^2+^) before boiling in 2× Laemmli loading buffer and being separated by SDS-PAGE.

### Immunoblotting and detection of cleaved spike proteins

Cell lysates were prepared from adherent syncytia and cell mixtures by direct lysis on ice in 2× reducing Laemmli loading buffer. Boiled lysate samples were separated using standard Tris-glycine sodium dodecyl sulfate-polyacrylamide gel electrophoresis (SDS-PAGE). Omni-Easy pre-mixed 7.5% polyacrylamide gels (Epizyme Scientific) were used for the detection of the cleaved S2′, while pre-cast 4%–12% gradient gels (Smart Lifesciences, SLE014) were used for NL63-HCoV N proteins. Separated proteins were then transferred onto 0.45 μm polyvinylidene difluoride (PVDF) membranes (Millipore) using a semi-dry transfer apparatus. All membranes were blocked in phosphate-buffered saline containing 0.1% Tween-20 (PBST) and 2.5% bovine serum albumin (BSA). Primary antibody incubations were performed at room temperature, followed by incubation with 1:5,000 diluted HRP-conjugated anti-mouse or anti-rabbit antibodies. Membrane blots were visualized using the PicoLight enhanced chemiluminescence solution (Epizyme Scientific) and digitally captured with a Tanon 5200 chemiluminescent imaging system with stacked molecular weight markers.

### Authentic virus infection assays and inhibitor timing assays

Authentic viruses were mixed with indicated concentrations of ConA at 37°C for 1 h as pre-treatment, before inoculating onto the seeded cells. The Caco-2 cells were infected with SARS-CoV-2 WT with MOI = 0.01 or hCoV-NL63 with a MOI = 0.2 at 34°C for 2 h. For pre-incubation timing of ConA, indicated concentrations of ConA were supplied at these timing points (−1, 0, 2, 12, and 24 h) prior to or after inoculation of hCoV-NL63. For hCoV-OC43-eGFP, Caco-2 cells were infected at 0.1 MOI. For SADS-CoV-DsRed, Huh-7-TMPRSS2 cells were infected at 0.3 MOI for 24 h.

Viral genome copies were determined using the infected Caco-2 cell supernatants 48 h after the SARS-CoV-2 and hCoV-NL63 infection. RT-PCR was performed using HiScript II One Step qRT-PCR SYBR Green Kit (Vazyme, Q221-01), in which samples were processed in duplicate using the following cycling protocol: 42°C for 5 min, 95°C for 10 s, followed by 45 cycles at 95°C for 5 s and 60°C for 34 s. The oligonucleotide sequences used for RT-PCR are targeted against the *E* gene of SARS-CoV-2 (sense: ACAGGTACGTTAATAGTTAATAGCGT, anti-sense: ATATTGCAGCAGTACGCACACA), or the *N* gene of hCoV-NL63 (sense: AGGACCTTAAATTCAGACAACGTTCT, anti-sense: GATTACGTTTGCGATTACCAAGACT) (38).

### Immunofluorescence assay

The Caco-2 cells were infected with SARS-CoV-2 (MOI = 0.01) or hCoV-NL63 (MOI = 0.2). 48 h after infection, cells were fixed with 4% paraformaldehyde and permeabilized with PBS supplemented with 0.2% Triton X-100. Cells were then incubated with anti-SARS-CoV-2 N (40143-R001, SinoBiological) or anti-hCoV-NL63 N Antibody (40641-T62, SinoBiological) at a 1:500 dilution, followed by a Cy3-conjugated goat anti-rabbit IgG polyclonal (HA1102, HUABIO) secondary antibody. Images of the cells were captured at endpoint using a 10x objective and a 12-bit monochrome complementary metal oxide semiconductor (CMOS) camera installed on the CKX53 inverted microscope (Olympus). The area of Caco-2 cell infection was quantified as image region areas using the High-content Imaging Systems (OperettaCLS, PerkinElmer).

### Focus-forming assay for hCoV-NL63, hCoV-OC43, and SADS-CoV

Determination of viral titer was based on the focus-forming assay (FFA). Viruses were serially diluted in DMEM. Caco-2 or Huh7-TMPRSS2 cells in 24-well plates were infected with viruses and inoculated at 34°C (hCoV-NL63, hCoV-OC43)/37°C (SADS-CoV) in 5% CO_2_ for 2 h with gentle rocking every 15 min. After removing the inoculant, plates were overlaid with 1% carboxymethylcellulose containing DMEM supplemented with 2% FBS. After further incubation for 72 h, cells were fixed with 4% formaldehyde for 2 h, before plaques were immunostained with rabbit anti-hCoV-NL63 N antibody (1:500 dilution, 40641-T62, SinoBiological), followed by HRP-conjugated goat anti-rabbit IgG (1:2,000 dilution, Jackson ImmunoResearch, 111-035-003) secondary antibody, visualized by TrueBlue substrate (KPL, 5510-0049). Alternatively, hCoV-OC43-GFP and SADS-CoV-DsRed were directly visualized by genetically encoded reporters. Viral titers were calculated and subsequently used as FFU per milliliter.

### Cell viability test

HEK293T, A549, and Caco-2 cells were seeded in 96-well plates before the test. The next day, cells were treated with serially diluted ConA in PBS and incubated at 37°C. After 24 h of treatment, culture supernatants were removed, and the remaining adherent cells were collected; 100 μL of CellTiter-Lumi Steady II luminescence detection reagent (C0058M, Beyotime) per well was directly added to cells according to the manufacturer’s protocol, and cell viability was determined using bioluminescence based on cellular ATP level on a Synergy H1 plate reader (Biotek).

### Negative-staining electron microscopy

For negative staining of SARS-CoV-2 spike and ConA, the complexes were obtained by incubating the S-6P-ECD and ConA at different molar ratios and were diluted to 0.1 mg/mL to check for the formation of large aggregates first and then further diluted 5–10 times to check the particle density. The appropriate protein distribution could be observed under 0.01–0.02 mg/mL for S alone; 3 μL samples were applied to carbon film 300 mesh copper grids (EMS) before being stained with 2% phosphotungstic acid (APExBIO) and examined using a Talos L120C (Thermo Scientific) transmission electron microscope at 120 kV.

### Statistical analysis

ANOVA and Student’s *t*-tests were used to analyze differences in mean values between groups using Prism 10 (GraphPad). All results are expressed as mean ± standard error of mean (SEM), and statistical significance was determined using Student’s *t*-test or one-way ANOVA with Sidak post-hoc multiple comparison analysis. Statistically significant *P*-values are indicated as *P < 0.05, **P < 0.005, ***P < 0.0005, ***P < 0.0001.

## Data Availability

This study did not generate any new data sets. Therefore, public data sharing is not applicable to this research.

## SUPPLEMENTAL MATERIAL

Supplemental material jvi.01679-25-s0001.docx Figures S1 to S5.

ASM does not own the copyrights to Supplemental Material that may be linked to, or accessed through, an article. The authors have granted ASM a non-exclusive, world-wide license to publish the Supplemental Material files. Please contact the corresponding author directly for reuse.
