# Supplementary material for: Concanavalin A targets phylogenetically conserved N-linked glycans on coronavirus spike proteins for broad-spectrum antiviral activity
Source: J Virol. 2026 Apr 27;100(5):e01679-25. doi: 10.1128/jvi.01679-25 (PMC13185596; doi:10.1128/jvi.01679-25)
Supplement: Supplemental material — Figures S1 to S5. [file jvi.01679-25-s0001.docx]

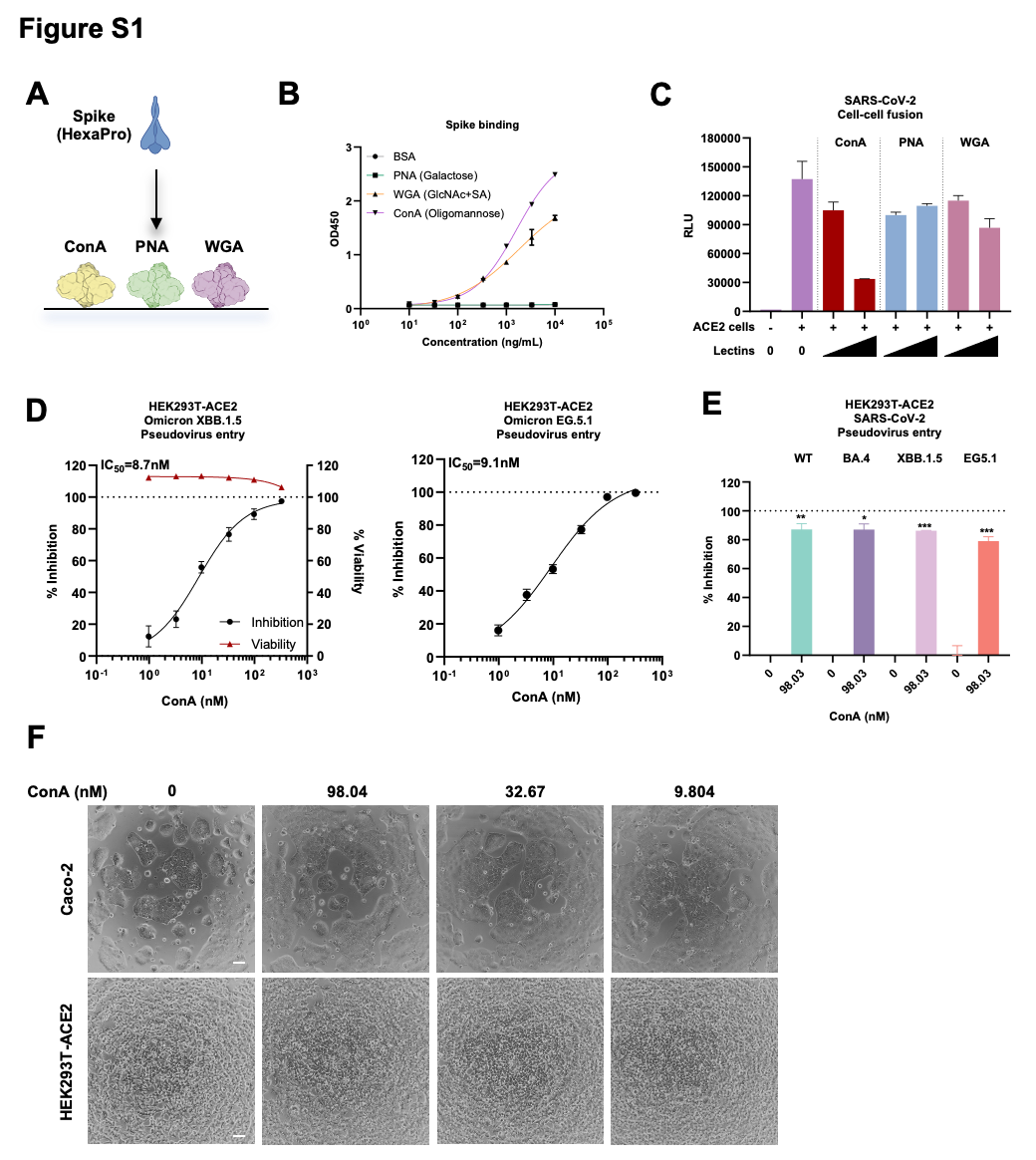


**Figure S1: Identification of ConA as a spike inhibitor**

1. Schematics of lectin-based ELISA showing binding of peanut agglutinin (PNA), wheat germ agglutinin (WGA) and concanavalin A (ConA) to the purified SARS-CoV-2 prefusion spike HexaPro ectodomain (S-6P-ECD).
2. ELISA showing binding of immobilized WGA and ConA, but not PNA to the purified S-6P-ECD. ELISA plates were coated with 1 µg/well PNA, WGA or ConA, bovine serum albumin (BSA) was used as a negative control. Data are representative of two replicates.
3. Luciferase activity (RLU) detected from SARS-CoV-2 WT spike-expressing cells, co-cultured with LoxP-luciferase control HEK293T or HEK293T-ACE2 cells for 16 hours in the absence or presence of 1 or 10 µg/mL PNA, WGA and ConA. Data are representative of three repeats and data points are represented as mean ± SEM.
4. Normalized IC_50_ curve (black) and cytotoxicity (red) of ConA for Omicron XBB.1.5 and EG.5.1 spike variants pseudovirus entry respectively on HEK293T-ACE2 cells. Data are representative of three repeats.
5. Normalized percentage inhibition of WT, BA.4, XBB.1.5 or EG.5.1 pseudovirus entry on HEK293T-ACE2 cells pre-treated without or with 98.03 nM (10 µg/mL) ConA. Data are representative of three repeats.
6. Representative brightfield images of Caco-2 and HEK293T cells without or with the treatment of 9.804 to 98.04 nM ConA for 24 h. The scale bar is representative of 100 μm.


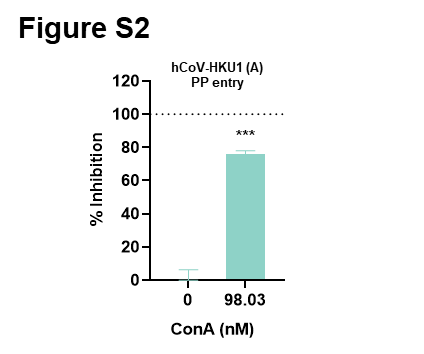


**Figure S2: Inhibition of pseudotype entry bearing the HKU1 (A) spike.**

Percentage inhibition of pseudovirus entry bearing the HKU1 (A) spike by 10 µg/mL ConA. Data are representative of three repeats. Statistical significance was determined using student’s t test. *P* values are indicated as ****P* < 0.0005.


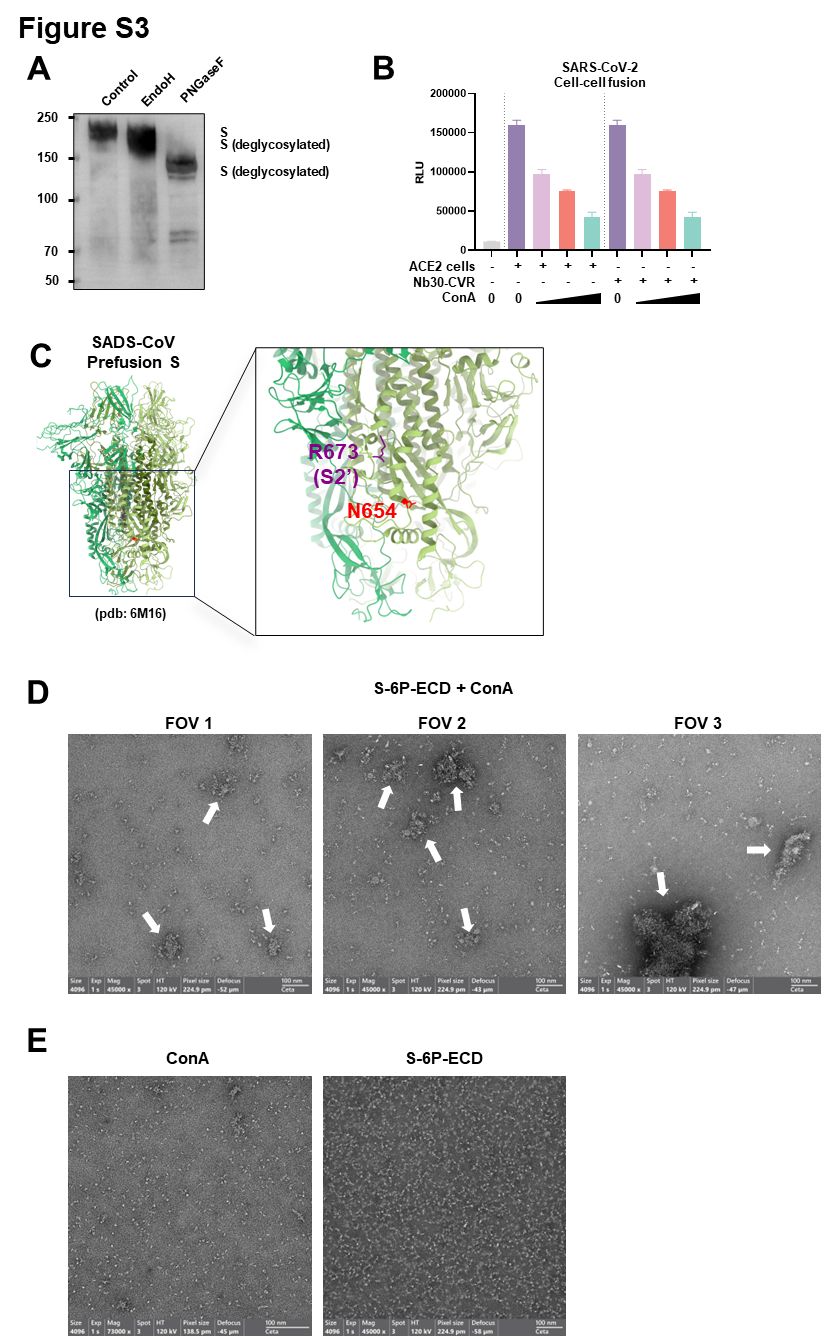


**Figure S3: N-glycosylation is associated with ConA-spike interaction.**

1. Immunoblots showing the full-length and deglycosylated S-6P-ECD treated without or with 10 units of EndoH or PNGaseF for 37^o^C for 4 h.
2. Luciferase activity (RLU) detected from SARS-CoV-2 WT spike-expressing cells, co-cultured with LoxP-luciferase control HEK293T, HEK293T transfected with human ACE2 or Nb30-CVR cells for 16 hours in the absence or presence of 0.98, 98.0 or 326.5 nM ConA. Data are representative of three repeats and data points are represented as mean ± SEM.
3. Structural representation of SADS-CoV spike N654 glycosylation site and the putative S2’ cleavage site R673 modeled on the prefusion spike (pdb: 6M16).
4. Representative electron micrographs from three different field of view (FOV) showing random aggregation of 20 μg/mL ConA mixed with 20 μg/mL S-6P-ECD at 1:1 ratio.
5. Representative electron micrographs from 20 μg/mL ConA or 20 μg/mL S-6P-ECD without mixing. Scale bar and magnifications are indicated on the micrographs.


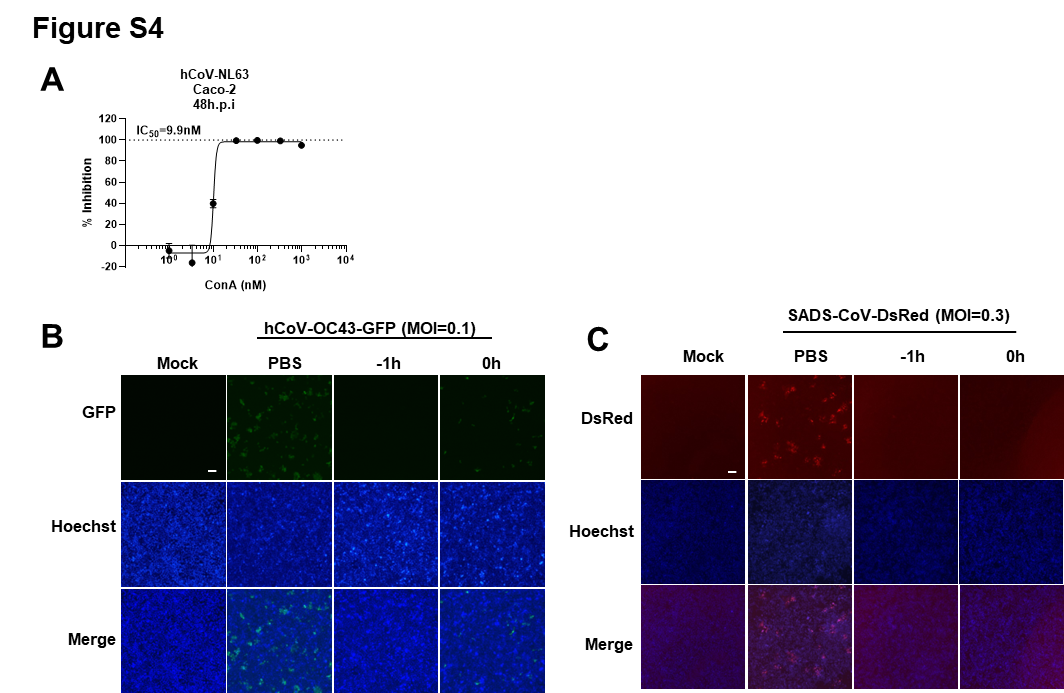


**Figure S4. ConA-mediated inhibition of authentic coronavirus infections by hCoV-OC43 and SADS-CoV.**

1. The IC_50_ of ConA for hCoV-NL63 in Caco-2 cells infected with MOI=0.2 hCoV-NL63 for 48 hours.
2. Representative fluorescent images of GFP and Hoescht33342 collected from 4% PFA-fixed Caco-2 cells infected with MOI=0.1 hCoV-OC43-GFP for 48 h in the presence of 20 µg/mL ConA. ConA was pre-incubated for 1 h or added immediately (0 h) prior to hCoV-OC43-GFP infection. Images are representative of two repeats, and the scale bar is representative of 100 μm.
3. Representative fluorescent images of DsRed and Hoescht33342 collected from 4% PFA-fixed Caco-2 cells infected with MOI=0.3 SADS-CoV-DsRed for 48 h in the presence of 20 µg/mL ConA. ConA was pre-incubated for 1 h or added immediately (0 h) prior to SADS-CoV-DsRed infection. Images are representative of two repeats, and the scale bar is representative of 100 μm.


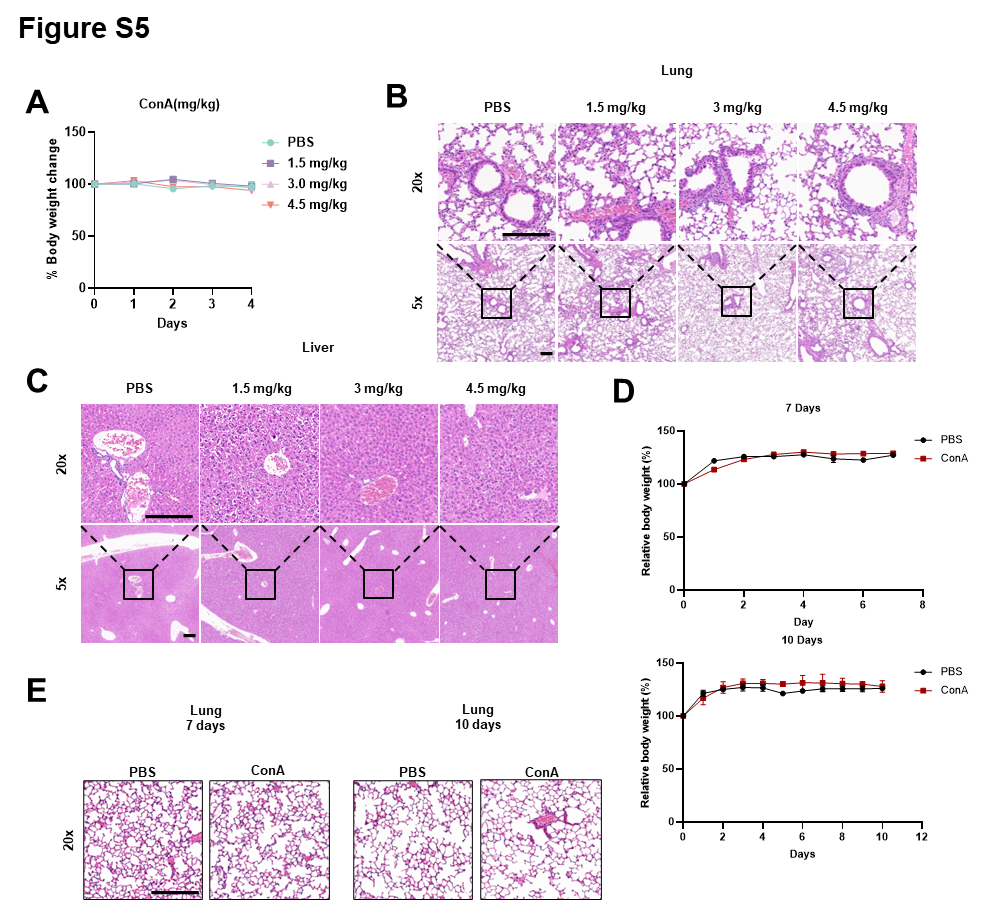


**Figure S5. Toxicity analysis of intranasally administered ConA in mice.**

1. The percentage of body weight changes in C57BL/6 mice (3 mice from each group) 4 days after intranasal delivery of vehicle control (PBS) or ConA at 1.5, 3.0 and 4.5 mg/kg. Body weights in grams taken at each measurement were normalized against the mice weight measure at day 0.
2. Representative H&E staining of lungs from C57BL/6 mice(n=3) collected at 4 days after intranasal delivery of various doses of ConA. Scale bars of 5x are 200μm, 20x are 50μm.
3. Representative H&E staining of lungs and livers from C57BL/6 mice(n=3) harvested 4 days after intranasal delivery of ConA. Scale bars of 5x are 200μm, 20x are 50μm.
4. The percentage of body weight changes in C57BL/6 mice (2 mice from each group) 7 days and 10 days after intranasal delivery of vehicle control (PBS) or ConA at 3.0 mg/kg. Body weights in grams taken at each measurement were normalized against the mice weight measure at day 0.
5. Representative H&E staining of lungs from C57BL/6 mice (n=2) collected at 7 and 10 days after intranasal delivery of 3 mg/kg ConA at 20x magnification. Scale bars are indicative of 100μm.
